# The alternative polyadenylation regulator CFIm25 promotes macrophage differentiation and activates the NF-κB pathway

**DOI:** 10.1186/s12964-025-02114-1

**Published:** 2025-02-28

**Authors:** Srimoyee Mukherjee, Atish Barua, Luyang Wang, Bin Tian, Claire L. Moore

**Affiliations:** 1 Department of Developmental, Molecular, and Chemical Biology, Tufts University School of Medicine, Boston, MA 02111 USA; 2 The Wistar Institute, Philadelphia, PA 19104 USA

**Keywords:** Alternative polyadenylation, Monocyte, Macrophage, CFIm25, Cell cycle, NUDT21

## Abstract

**Background:**

Macrophages are required for development and tissue repair and protect against microbial attacks. In response to external signals, monocytes differentiate into macrophages, but our knowledge of changes that promote this transition at the level of mRNA processing, in particular mRNA polyadenylation, needs advancement if it is to inform new disease treatments. Here, we identify CFIm25, a well-documented regulator of poly(A) site choice, as a novel mediator of macrophage differentiation.

**Methods:**

CFIm25 expression was analyzed in differentiating primary human monocytes and monocytic cell lines. Overexpression and depletion experiments were performed to assess CFIm25’s role in differentiation, NF-κB signaling, and alternative polyadenylation (APA). mRNA 3’ end-focused sequencing was conducted to identify changes in poly(A) site use of genes involved in macrophage differentiation and function. Cell cycle markers, NF-κB pathway components, and their targets were examined. The role of CFIm25 in NF-κB signaling was further evaluated through chemical inhibition and knockdown of pathway regulators.

**Results:**

CFIm25 showed a striking increase upon macrophage differentiation, suggesting it promotes this process. Indeed, CFIm25 overexpression during differentiation amplified the acquisition of macrophage characteristics and caused an earlier slowing of the cell cycle, a hallmark of this transition, along with APA-mediated downregulation of cyclin D1. The NF-κB signaling pathway plays a major role in maturation of monocytes to macrophages, and the mRNAs of null, TBL1XR1, and NFKB1, all positive regulators of NF-κB signaling, underwent 3’UTR shortening, coupled with an increase in the corresponding proteins. CFIm25 overexpression also elevated phosphorylation of the NF-κB-p65 transcription activator, produced an earlier increase in the NF-κB targets p21, Bcl-XL, ICAM1 and TNF-α, and resulted in greater resistance to NF-κB chemical inhibition. Knockdown of Tables 2 and TBL1XR1 in CFIm25-overexpressing cells attenuated these effects, reinforcing the mechanistic link between CFIm25-regulated APA and NF-κB activation. Conversely, depletion of CFIm25 hindered differentiation and led to lengthening of NFKB1, TAB2, and TBL1XR1 3’ UTRs.

**Conclusions:**

Our study establishes CFIm25 as a key mediator of macrophage differentiation that operates through a coordinated control of cell cycle progression and NF-κB signaling. This linkage of mRNA processing and immune cell function also expands our understanding of the role of alternative polyadenylation in regulating cell signaling.

**Supplementary Information:**

The online version contains supplementary material available at 10.1186/s12964-025-02114-1.

## Background

Monocytes and macrophages are members of the mononuclear phagocyte system, a component of innate immunity [1]. Monocytes proliferate in response to infection and injury, and after rushing to the site of interest, mature into active macrophages that can combat invading pathogens or promote tissue repair [2]. In order to manipulate macrophages to be more effective at these roles, we need to better understand how gene expression is regulated during their differentiation.

Alternative polyadenylation (APA) is a widespread mechanism that regulates mRNA and protein isoform expression by determining the position where poly(A) is added to the 3’ end of mRNA transcripts [3–5]. APA often results in mRNA isoforms with the same coding sequence but different 3’ UTRs lengths, with downstream effects on mRNA stability, translatability, localization, and RNA-seeded protein/protein interactions. In contrast, intronic APA alters the coding sequence. We have already documented APA as an important phenomenon in the complex process of monocyte-macrophage differentiation and showed a role for the Cleavage/Polyadenylation (C/P) protein CstF64 in promoting this differentiation [6]. However, many global APA regulators have been identified [7, 8], and it is likely that other proteins besides CstF64 will influence macrophage differentiation through APA. A possible candidate to modulate this transition is the mRNA 3’end processing factor CFIm25 (CPSF5/NUDT21), which is known to be a principal regulator of 3′ UTR length in many cell fate transitions [9–15]. However, its role in macrophage differentiation has not yet been investigated. In our previous study, we have found that compared to other C/P proteins, CFIm25 increased at a time when the first signs of macrophage differentiation were starting to appear [6]. Based on this upregulation, we hypothesized that CFIm25 plays a critical role in the differentiation process and influences important pathways that govern macrophage fate.

To perform its function as an APA regulator, CFIm25 binds specifically to a UGUA motif within the pre-mRNA as a dimer [16, 17] and associates with either the CFIm68 (CPSF6) or CFIm59 (CPSF7) subunit to form an active CFIm complex [18]. Mechanistically, CFIm25 exerts its effect on cell fate by inducing a widespread switch of APA patterns in hundreds of transcripts [14]. Multiple studies have revealed that CFIm25 levels affect the proliferation of many types of tumor cells, with CFIm25 being mostly tumor-suppressive, in part by causing lengthening of transcripts of the cell cycle gene CCND1 [14, 19, 20]. CFIm25 also has antifibrotic properties, and its down regulation amplifies lung, skin, and liver fibrosis [21–23]. CFIm25 blocks the generation of pluripotent stem cells [15], is needed for the differentiation of embryonic stem cells into the ectodermal lineage, and promotes differentiation of neutrophils from a myeloid progenitor cell line [15].

In the current study, we show that CFIm25 overexpression accelerates macrophage differentiation and provide new insights into the molecular regulation of this important cell fate transition. In particular, CFIm25 regulates expression of genes in the NF-κB pathway, which plays a major role in maturation of monocytes to macrophages and protecting these cells from apoptosis [24–27]. CFIm25 modulates the APA and protein levels of the NF-κB regulators TAB2, TBL1XR1, and NFKB1, which are known to enhance NF-κB activation by promoting TAK1 kinase function and NF-κB target gene transcription [28–31]. These changes were correlated with increased phosphorylation of the NF-κB p65 subunit, upregulation of NF-κB targets (p21, Bcl-XL, ICAM1, and TNF-α), and resistance to NF-κB inhibition. Knockdown of TAB2 and TBL1XR1 in CFIm25-overexpressing cells reduced these effects, reinforcing the mechanistic link between CFIm25-mediated APA and NF-κB activation. Our findings identify a new function of CFIm25 in orchestrating the macrophage differentiation program through activation of NF-κB signaling and highlight the role of APA in mediating this transition.

## Materials and methods

### Cell culture, treatment, and microscopy

All cells were maintained in RPMI 1640 medium supplemented with 2 mM L-glutamine and 10% heat-inactivated fetal bovine serum (FBS) at 37 °C in a humidified atmosphere of 5% CO_2_. Differentiation of the HL-60 (ATCC^®^ CCL-240) and THP-1 (ATCC^®^ TIB-202) human monocytic cells into macrophages was performed by treatment with 3 nM phorbol-12-myristate-13-acetate (PMA; Sigma-Aldrich) for up to 24 h. Cells were cultured in 6-well plates at a density of 1 × 10^6^ cells/well. For the NF-κB inhibitor assays, HL-60 and THP-1 cells in both control and overexpressing CFIm25 groups were treated with 5 µM BI605906 or BAY-11-7082, and after 24 h, the cells were treated with PMA in fresh media without inhibitor, followed by subsequent analyses.

To culture human primary monocytes, human whole blood in K3-EDTA was purchased from Vitrologic (Charleston, SC). Collection and use of blood were conducted according to the guidelines of IRB (Institutional Review Board)-approved informed consent and protocols. Human Peripheral Blood Mononuclear Cells (PBMCs) were isolated from whole blood by the Ficoll-Paque density centrifugation method [32]. Then, monocytes were enriched by negative selection using EasySep™ Human Monocyte Isolation Kit (Stem Cell Technologies) according to manufacturer’s protocol. The primary human monocytes were differentiated with 50 ng/ml recombinant human M-CSF (Peprotech) for 5–7 days to obtain macrophages. For monitoring morphological changes, cells were cultured in 6-well plates at a density of 1 × 10^6^ cells/well, after which they were examined and photographed using an EVOS FL fluorescence phase contrast microscope (Thermo Fisher Scientific). All results were obtained from three independent experiments.

### Attachment, proliferation and viability assays

HL-60 and THP-1 cells were either untreated or treated with PMA and left for the indicated times. After that, unbound cells were collected from the plate by washing gently with phosphate-buffered saline (PBS) and collected for counting (denoted as “suspended”). The attached cells were collected by scraping to release them from the plate. Cells were then stained with trypan blue and counted in a hemocytometer and represented as percentage of suspended or attached cells over total (suspended + attached). The figures are an average of three independent observations. The number of live cells before and after differentiation were calculated by trypan blue exclusion to see how well the cells proliferated in each condition. For the viability assay, we used resazurin for a fluorescent assay that detects cellular metabolic activity. The blue nonfluorescent resazurin reagent is reduced to highly fluorescent resorufin by dehydrogenase enzymes in metabolically active cells. This conversion only occurs in viable cells and thus, the amount of resorufin produced is proportional to the number of viable cells in the sample. The resorufin formed in the assay is quantified by measuring the relative fluorescence units (RFU) at 590–620 nm.

### Cell cycle analysis by DNA content

Cell cycle analysis was carried out using propidium iodide staining methods [33]. Briefly, 1 × 10^6^ cells were pelleted at 400 g, resuspended in 1 ml of ice-cold 70% ethanol, and left at 4 °C for at least 24 h (up to 2 months). On the day of data acquisition, cells were spun at 400 g for 5 min and washed twice in PBS. The pellet was resuspended in 300 µl of the Cell Cycle Staining Solution [38 mM sodium citrate, 10 mg/ml pancreatic RNase A (Signa-Aldrich), 68 mM propidium iodide (Sigma Aldrich)]. Samples were acquired on a FACS Calibur or BDLSRII cytometer (BD Biosciences, Franklin Lakes, NJ, United States) using a 610 nm laser. Results were analyzed using Cell Quest Pro, FACS Divia, and FlowJo software version 10.0.8r1 (Flow Jo LLC, Ashland, OR, United States), gating single viable cells and excluding doublets. A total of 10,000 events were acquired for each sample. The histogram of the area of the propidium iodide channel was examined using the cell cycle option of the respective software, choosing the Watson (pragmatic) type of analysis [34].

### Western blot analysis

Western blots were performed with total cell extracts of untreated and differentiated cells. For preparation of extract, RIPA buffer (20 mM Tris-HCl pH 7.5, 150 mM NaCl, 1 mM Na_2_EDTA, 1 mM EGTA, 1% NP-40, 1% sodium deoxycholate, 2.5 mM sodium pyrophosphate, 1 mM β-glycerophosphate, 1 mM Na_3_VO_4_ and 1 µg/ml leupeptin) was added to the cell pellet, followed by incubation on ice for 15 min, centrifugation at 25,000 rcf for 10 min at 4 °C, and supernatant collection. Protein concentration was measured with the BCA reagent (Pierce, Thermo Fisher Scientific), and 50–80 µg protein was separated on a 10% polyacrylamide-SDS gel and transferred to PVDF membrane. Ponceau Red staining was done for total protein staining of blots. The membrane was then cut into segments to enable probing for multiple proteins from the same blot. For overexpression and knockdown experiments, control and treated samples were run on the same gel. The membrane was then blocked for 1 h in 5% non-fat dried milk in TBS-T (Tris Buffer Saline with Tween-20, i.e., 0.05% Tween 20 in 1X TBS) buffer followed by rocking overnight at 4˚C with primary antibodies (antibody sources provided in Supplementary Table 1). Unbound antibodies were removed by 4 × 5 min washes with TBS-T buffer. The membrane was then incubated with an HRP-conjugated secondary antibody at a dilution of 1:5000 for 1 h at room temperature followed by 3 × 5-min washes with TBS-T and a final wash with TBS. The blot was developed with SuperSignal™ West Pico PLUS or Femto Chemiluminescent Substrate (Thermo Fisher Scientific), visualized with a ChemiDoc XRS + System (Bio-Rad), and quantitated using Image J software [35]. GAPDH, β-actin and Lamin B1 were used as control for individual western blots because their levels did not change upon differentiation. Pre-stained protein markers were used as internal molecular mass standards, and each western blot was performed in three biological replicates of each time point of the differentiation process.

### Flow cytometry analysis for macrophage markers

Surface marker analysis of macrophages was performed using flow cytometry. After differentiation, cells were recovered from culture plates by gentle scraping. Cells were washed in a solution of 1X Phosphate Buffered Saline (137 mM NaCl, 2.7 mM KCl, 8 mM Na_2_HPO4, and 2 mM KH_2_PO4), 1% BSA, 0.01 M NaN_3_ and incubated with Human TruStain FcX™ (Fc blocker, Biolegend) for 15 min to block Fc receptors and reduce nonspecific binding. Thereafter, cells were stained with 1:20 of PE-tagged CD38 or PE-tagged CD11b (macrophage markers) antibodies for 30 min on ice. Cells were fixed with buffer (Cytofix, Becton-Dickinson Biosciences), according to the manufacturer’s protocol. Samples were acquired on a BD LSRII flow cytometer, and data were analyzed using FACS diva or FlowJo. For analysis, samples were gated on light scattering properties to exclude dead cells and debris. Unstained control samples were used to determine the level of background fluorescence. The background fluorescence or autofluorescence was nullified by plotting the untagged cells against the fluorescent tag and any scattering parameters, and the fluorescent tag was plotted against forward scatter.

### RNA sequencing by QuantSeq

Library preparation of total RNA was carried out by using the Lexogen 3′ mRNA-Seq QuantSeq FWD kit according to manufacturer’s instructions. Library preparation, quality control, and sequencing were carried out by Admera Health (South Plainfield, NJ, USA). cDNA libraries were sequenced on an Illumina HiSeq machine (2 × 150 nt) at Admera Health.

### APA analysis

The reverse read (read 2) of QuantSeq FWD was used for APA analysis. Briefly, adapter and poly(T) regions were trimmed and trimmed reads were aligned by using STAR-2.7.7a to the human genome sequence (hg19). Only the alignments to PAS regions annotated by the PolyA_DB v3 database (-100 to + 25 nt around each PAS) were kept. The last aligned position (LAP) of each mapped read was compared to PolyA_DB-annotated-PASs [36], allowing ± 24 nt flexibility. Matched reads were considered PAS-supporting (PASS) reads, which were used for further APA analysis. The abundance of transcripts associated with a given PAS was based on the number of PASS reads normalized to the total number of PASS reads per sample, yielding a reads per million (RPM) value. UCSC genome browser tracks were generated by bedtools v2.31.0 [37] and bedGraphToBigWig [38].

For 3′ UTR APA analysis, we used DEXSeq analysis [39], where the two PASs with the highest usage levels in the 3′ UTR of the last exon were compared. One was named proximal PAS (pPAS) isoform, and the other distal PAS (dPAS) isoform. The difference in log_2_[reads per million (RPM) ratios] of the two PAS isoforms between two samples (control and CFIm25 overexpression) was defined as relative expression difference (RED). Significant APA events were those with RED > log_2_(1.2) or < − log_2_(1.2) and *p* < 0.05.

### RT-qPCR analysis

Total RNA extraction was carried out on 2 × 10^6^ cells using Trizol according to manufacturer’s protocol. 1.5 µg of RNA was subjected to reverse transcription using oligo dT primer and Superscript III reverse transcriptase. The cDNA was amplified by qPCR with specific primers (Supplementary Table 2) using the C1000™ thermal cycler with CFX96 Touch Real-Time PCR Detection System (Bio-Rad Laboratories). The relative expression of genes was analyzed quantitatively by the ΔΔC_t_ method. ACTB RNA was used as the normalization control for RT-qPCR-based RNA expression analyses because its level did not change upon differentiation according to the QuantSeq datasets. Primers for total or long transcripts were designed according to the PAS annotation featured in the PolyA_DB database and the PAS that was affected by PMA treatment (as observed in the UCSC genome browser representation of the QuantSeq data).

### Lentivirus construction, SiRNA and cell treatment

The OE-CFIm25 lentivirus vector was a kind gift from Shervin Assassi, University of Texas Health Science Center at Houston, TX [23]. It was generated by cloning the coding sequence (CDS) of human CFIm25 into the pLV-EF1a-IRES-Puro Vector (Addgene) that contains an EF-1a promoter upstream of an IRES element to co-express the puromycin marker. The CFIm25 CDS was inserted between the EF-1a promoter and IRES, which allows the expression of CFIm25 and the puromycin marker from a single mRNA sequence. The vector backbone without an insert was used as OE-control. For CFIm25, TAB2 and TBL1XR1 KD, ON-TARGETplus SMARTpool human siRNAs against each were purchased from Horizon Discovery Biosciences Limited.

### Lentiviral transfection

The recombinant plasmids were co-transfected with the components of Dharmacon™ Trans-Lentiviral packaging kit into HEK293FT cells using FuGENE^®^ HD (Promega Corp.) according to manufacturer’s protocol. Transfection of HEK293FT cells was carried out in 6-well plates when the cells were 80–85% confluent, and transfection media were changed after 16 h. The recombinant lentiviruses were harvested at 48 h post-transfection, spun at 1250 rpm for 5 min and filtered by a 0.45 μm filter to remove cells and debris. Purified viruses were used for infecting monocytic cells. 1 × 10^6^ target cells were seeded in 2 mL of media per well in a 6-well plate and cultured overnight. Lentiviral particles (2 mL per well) were added the next day to the cells in culture media containing 10 µg/mL polybrene for efficient infection. Selection of cells stably expressing OE-control and OE-CFIm25 started 72 h post-transfection. Growth medium was replaced with fresh selection medium containing 1 µg/mL of puromycin. Puromycin-containing medium was refreshed every 2–3 days, and selection was completed after approximately 1 week, after which clones were expanded for 2 more weeks and then frozen for later use.

### siRNA transfection

For all KDs, cells are seeded, allowed to rest for 1 h, then transfected with 50 nM of siRNA pool with jetOPTIMUS^®^ transfection reagent (Polyplus). This was followed by a 6-hour resting time for the cells to take in RNA and recover. Cells are then distributed evenly into six plates that are used for the different time points. Cells are harvested for the 0 h and PMA is added to the remaining plates for the other time points. The time gap between seeding transfected cells and PMA treatment is 7 h.

### ELISA for TNF-α

The BioLegend^®^ Legend Max™ kit was used to perform ELISA according to manufacturer’s protocol. In this sandwich ELISA, a human TNF-α specific monoclonal antibody is precoated on a 96-well strip-well plate. Supernatants from PMA-treated HL-60 and THP-1 cells are used to quantify the amount of expressed TNF-α, which was represented as pg/mL.

### Statistical analysis

Experiments were performed in at least three independent sets. Data are presented as mean ± SE. Statistical analysis was performed using GraphPad Prism 6.01 (GraphPad Software Inc., La Jolla, CA, USA). Two-way analysis of variance (ANOVA) was performed to determine the significance between the groups. Considerations were * = *P* ≤ 0.05; ** = *P* ≤ 0.01; *** = *P* ≤ 0.001. A p value < 0.05 was considered significant.

## Results

### Macrophage differentiation is accompanied by enhanced CFIm25 expression

**Fig. 1:**
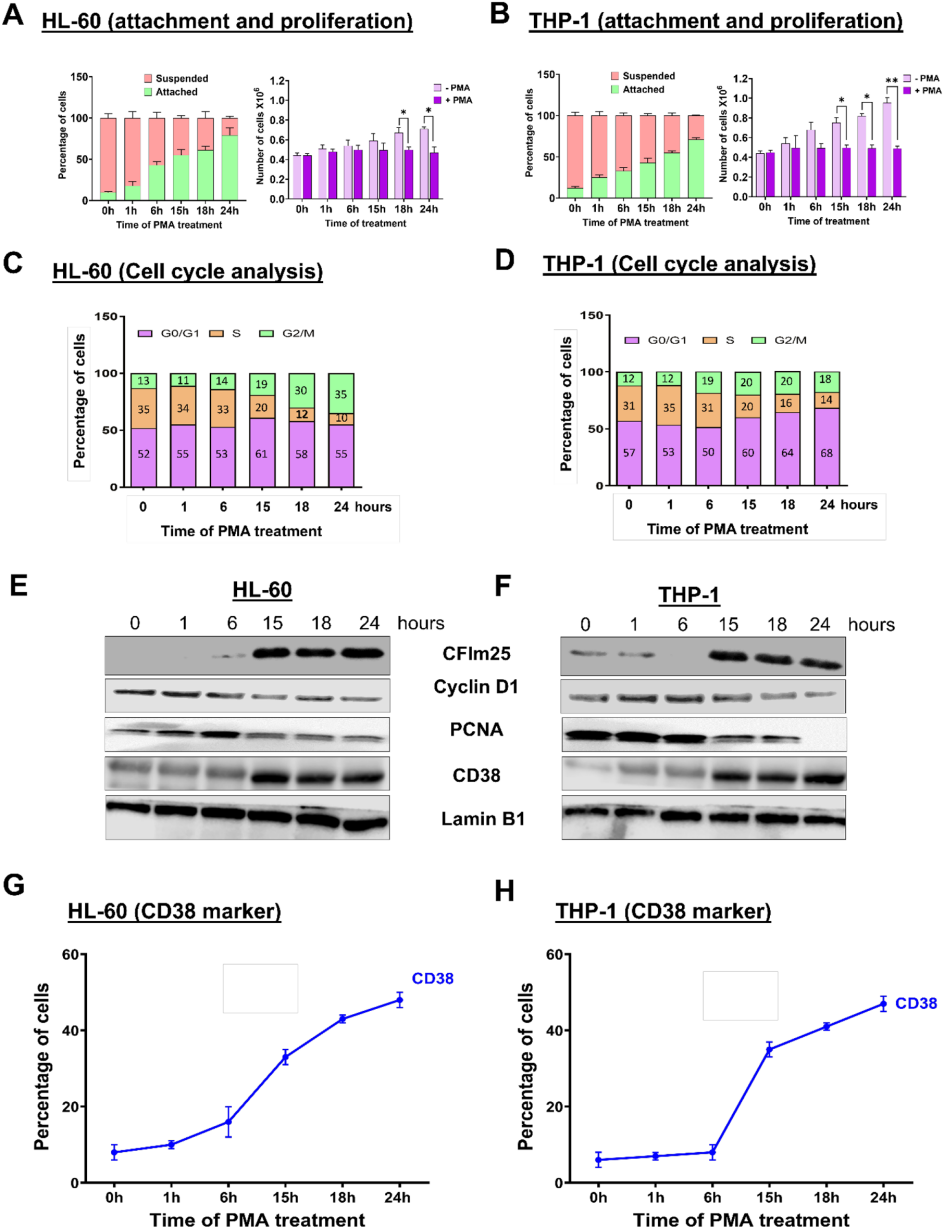
PMA treatment leads to differentiation and enhanced CFIm25 expression. (**A** and **B**) Attachment and proliferation assays in (**A**) HL-60 and (**B**) THP-1 cells. For the attachment assay (left), cells were treated with 3 nM PMA for 0, 1, 6, 15, 18 and 24 h and live cells were visualized and counted by the Trypan blue exclusion assay. The graph presents the percentage of cells that are suspended or attached. For the proliferation assay (right), the number of cells before and after differentiation depicts the absolute cell number in millions, compared to normal cell proliferation in the absence of PMA. The figure represents mean ± SE from three independent experiments. P value < 0.05 was considered significant, where * = *P* ≤ 0.05; **= *P* ≤ 0.01. (**C** and **D**) Cell cycle analysis for the HL-60 (**C**) and THP-1 (**D**) time courses. Cell cycle progression was determined by PI staining followed by flow cytometry at the indicated times after PMA treatment. Quantitative measurement of cell cycle phase is presented as a stacked bar graph. Numbers in each column represent the percentage of cells in the specified phase. Data is representative of at least three biological replicates. (**E** and **F**) Analysis of protein levels during HL-60 (**E**) and THP-1 (**F**) time courses. Western blot of the C/P protein CFIm25, the cell cycle marker cyclin D1, the proliferation marker PCNA, and the macrophage marker CD38, in cells treated with PMA for the indicated times, where Lamin B1 serves as the loading control. (**G** & **H**) CD38 levels by flow cytometry of HL-60 (**G**) and THP-1 (**H**) cells. The expression of CD38 over time is shown as % CD38 + cells. The figure represents mean ± SE from three independent experiments

To study the impact of alternative polyadenylation (APA) in macrophage differentiation, we used two well-established monocytic cell lines, HL-60 and THP-1 [40, 41], which can be differentiated to macrophages by treatment with PMA [42–44]. Treatment of both cell lines was done for a time course (0–24 h), and as observed previously [6, 45], these cells, which normally grow in suspension, displayed increased adherence to the culture dish and no longer proliferated after PMA treatment (Fig. 1A and B). By 24 h, most cells showed other macrophage-like phenotypes such as increased cell size and appearance of filopodia (data not shown).

Cells usually slow down their cell cycle progression while differentiating into distinct cell types [46]. By examining the cell cycle distribution of HL-60 and THP-1 cells during differentiation with flow cytometry, we found that for both cell types, the response to PMA involved slowing of cell cycle progression, with the number of cells in the S phase starting to decrease after 6h (Fig. 1C and D, and Supplementary Fig. 1A and B). Consistent with decreased proliferation, the levels of Proliferating Cell Nuclear Antigen (PCNA), a well-known proliferation marker, and Cyclin D1 (CCND1), a protein which modulates the transition from G1 to S phase, both decreased in expression over the differentiation time course of HL-60 and THP-1 cells (Fig. 1E-F). Macrophage differentiation is also associated with increased expression of macrophage markers [47]. By western blot analysis, we observed an increase in the macrophage marker CD38 [48] (Fig. 1E-F). Flow cytometry analysis showed that CD38 surface expression also increased (Fig. 1G-H and Supplementary Fig. 1C-D).

We recently showed that the cellular changes occurring during macrophage differentiation were accompanied by an overall increase in the expression of the C/P complex proteins, including CFIm25 [6]. In agreement with our earlier work, PMA treatment in the present study increased the levels of CFIm25 in both HL-60 and THP-1 cells at a time frame similar to the changes observed in cell cycle progression and macrophage marker expression (Fig. 1E-F). We also examined CFIm25 levels in human primary blood monocytes induced to differentiate into macrophages with M-CSF. After M-CSF addition, the monocytes attached to the plates and exhibited morphological changes consistent with efficient differentiation as well as increased expression of macrophage surface markers CD16, HLA-DRA, and ICAM1 (Supplementary Fig. 1E-F). We found a large increase in CFIm25 protein level after seven days of M-CSF exposure (Supplementary Fig. 1G). This data demonstrates that the increase in CFIm25 protein that we observed during differentiation of monocytic cell lines is also a feature of primary human monocytes and not a model-specific phenomenon. As described in the Introduction, the CFIm25 protein is known to promote alternative polyadenylation with functional consequences in many cellular transitions, and our goal in this study was to determine the role of CFIm25 in macrophage differentiation.

### CFIm25 overexpression promotes macrophage differentiation and expedites the cell cycle exit

**Fig. 2:**
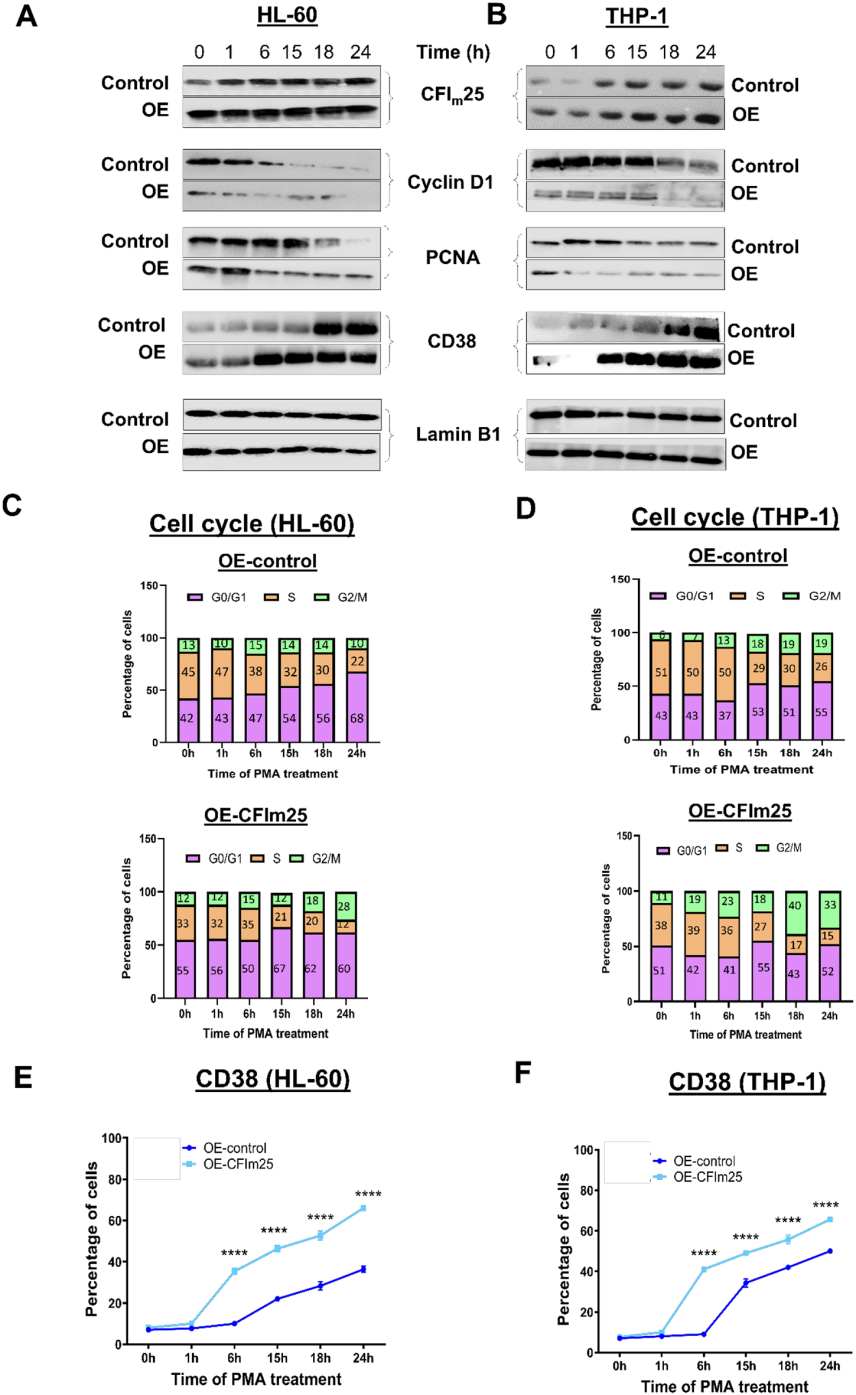
CFIm25 overexpression promotes the differentiation to macrophages and expedites the cell cycle exit. (**A** and **B**) Western blot analysis of CFIm25, cyclin D1, PCNA and CD38 for HL-60 (**A**) and THP-1 (**B**) overexpressing CFIm25 (OE) with respect to control, at the indicated times of PMA treatment. Lamin B1 serves as the loading control. (**C** and **D**) Cell cycle analysis for (**C**) HL-60 or (**D**) THP-1 cells overexpressing CFIm25 and treated with PMA, performed as described in Fig. 1. (**E** and **F**) CD38 levels by flow cytometry of (**E**) HL-60 and (**F**) THP-1 control cells (dark blue) or cells overexpressing CFIm25 (light blue). The expression of CD38 over time is shown as percentage of CD38 + cells. Data is representative of at least three biological replicates and is plotted as mean ± SE. P value < 0.05 was considered significant, where **** = *P* ≤ 0.0001

To find out if overexpression of CFIm25 in PMA-treated cells would accelerate the appearance of differentiation phenotypes, we overexpressed CFIm25 in both HL-60 and THP-1 cells with the help of lentiviral constructs (Fig. 2A and B). There was a marked increase in attachment at 6h with no change in cell viability in CFIm25 overexpressing cells compared to controls (Supplementary Fig. 2A and B). An earlier decrease in Cyclin D1 and PCNA further indicated enhanced differentiation (Fig. 2A and B). In accordance with this finding, cells overexpressing CFIm25 had fewer cells in S phase over the differentiation time course, indicating stronger slowing of cell cycle progression for both HL-60 and THP-1 cells compared to the controls (Fig. 2C and D and Supplementary Fig. 2C and D). An enhanced differentiation is also evident from an earlier increase (at 6 h) in the macrophage marker CD38 as demonstrated by western blot (Fig. 2A and B) and an earlier and stronger increase in surface CD38 as measured by flow cytometry (Fig. 2E-F and Supplementary Fig. 2E-F). In summary, overexpression of CFIm25 slows cell cycle progression as evident from flow cytometry and repression of PCNA and Cyclin D1 and enhances expression of a surface marker that distinguishes monocytes from macrophages.

### CFIm25 depletion suppresses features of differentiation

**Fig. 3:**
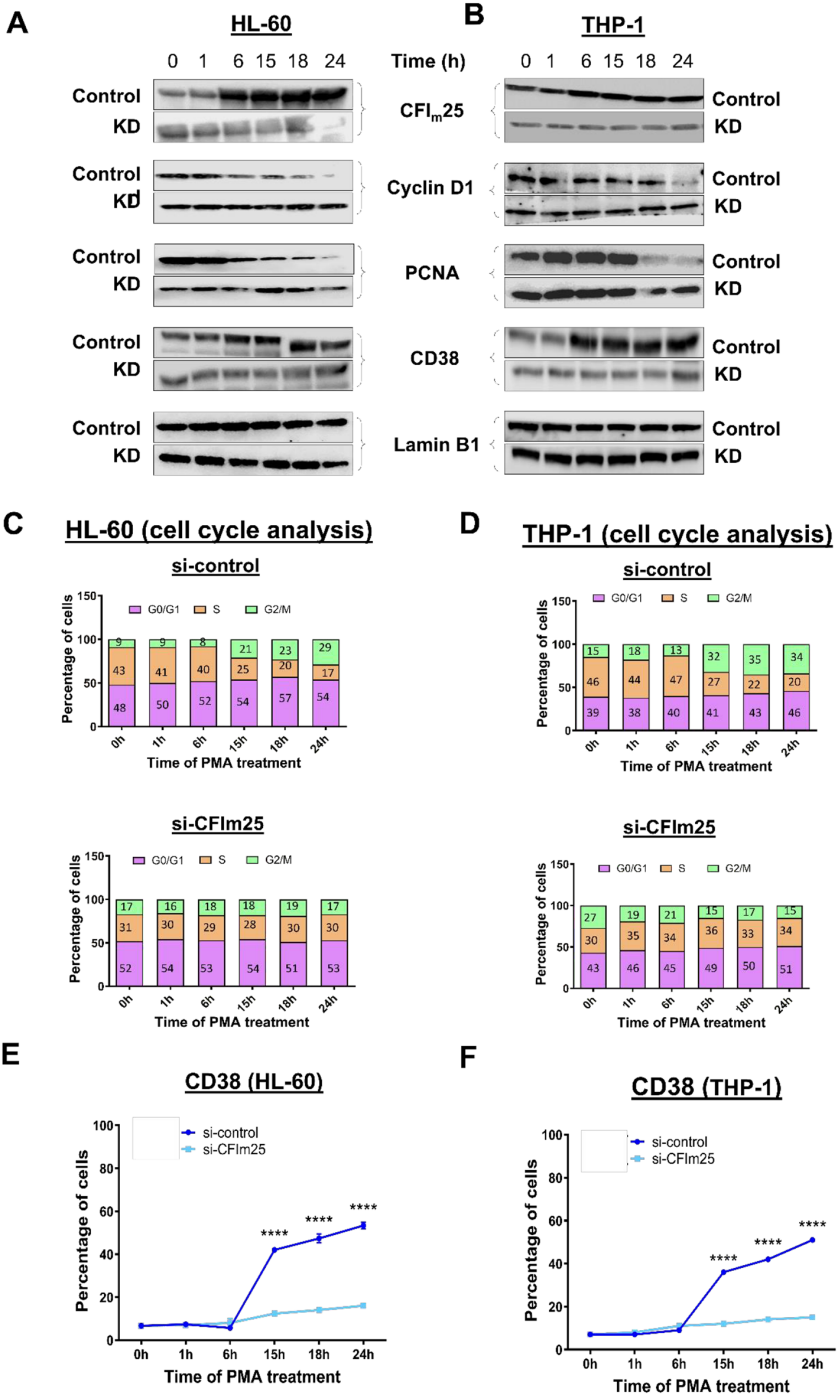
CFIm25 knockdown delays slowing of cell cycle and differentiation to macrophages. (**A** and **B**) Western blots for proteins in HL-60 (**A**) and THP-1 (**B**) cells. Western blot analysis of CFIm25, cyclin D1, PCNA and CD38 at the indicated times of PMA treatment for cells knocked down with siRNA against CFIm25 (KD) with respect to si-control. Lamin B1 serves as the loading control for both the cells. (**C** and **D**) Cell cycle analysis after knockdown of CFIm25 in HL-60 (**C**) and THP-1 (**D**) cells treated with PMA. Cell cycle analysis was performed as described in Fig. 1. (**E** and **F**) CD38 levels by flow cytometry of HL-60 (**E**) and THP-1 (**F**) control cells and cells depleted of CFIm25. The expression of CD38 over time is shown as % CD38 + cells. Data is representative of at least three biological replicates and is plotted as mean ± SE. P value < 0.05 was considered significant, where **** = *P* ≤ 0.0001

To determine if CFIm25 depletion has effects on differentiation opposite to that of overexpression, CFIm25 in HL-60 and THP-1 cells was knocked down by transfecting pooled siRNAs against this C/P protein. There was a considerable decrease in CFIm25 protein expression, indicating efficient knockdown (Fig. 3A and B). After PMA treatment and CFIm25 knockdown (KD), these cells displayed decreased attachment but no change in viability (Supplementary Fig. 3A and B). For both cell lines, CFIm25 knockdown blocked the slowing of the cell cycle seen in control cells, with no drop in the number of cells in S phase (Fig. 3C and D, and Supplementary Fig. 3C and D). In addition, Cyclin D1 levels remained high throughout the time course in the KD cells compared to controls, and PCNA levels did not decline as much at the later time points (Fig. 3A and B). By western blot, the macrophage marker CD38 failed to increase over the time course in comparison to the control cells (Fig. 3A and B). By flow cytometry, knockdown strongly repressed the increase of surface CD38 seen in the control cells (Fig. 3E-F and Supplementary Fig. 3E-F). These findings show that depleting CFIm25 leads to a strong delay in differentiation of monocytes to macrophages. Together, the CFIm25 overexpression and depletion experiments indicate that the normal increase in CFIm25 during differentiation expedites the transition of monocytes to macrophages.

### Alternative polyadenylation of CCND1 May contribute to the CFIm25-mediated cell cycle regulation

**Fig. 4:**
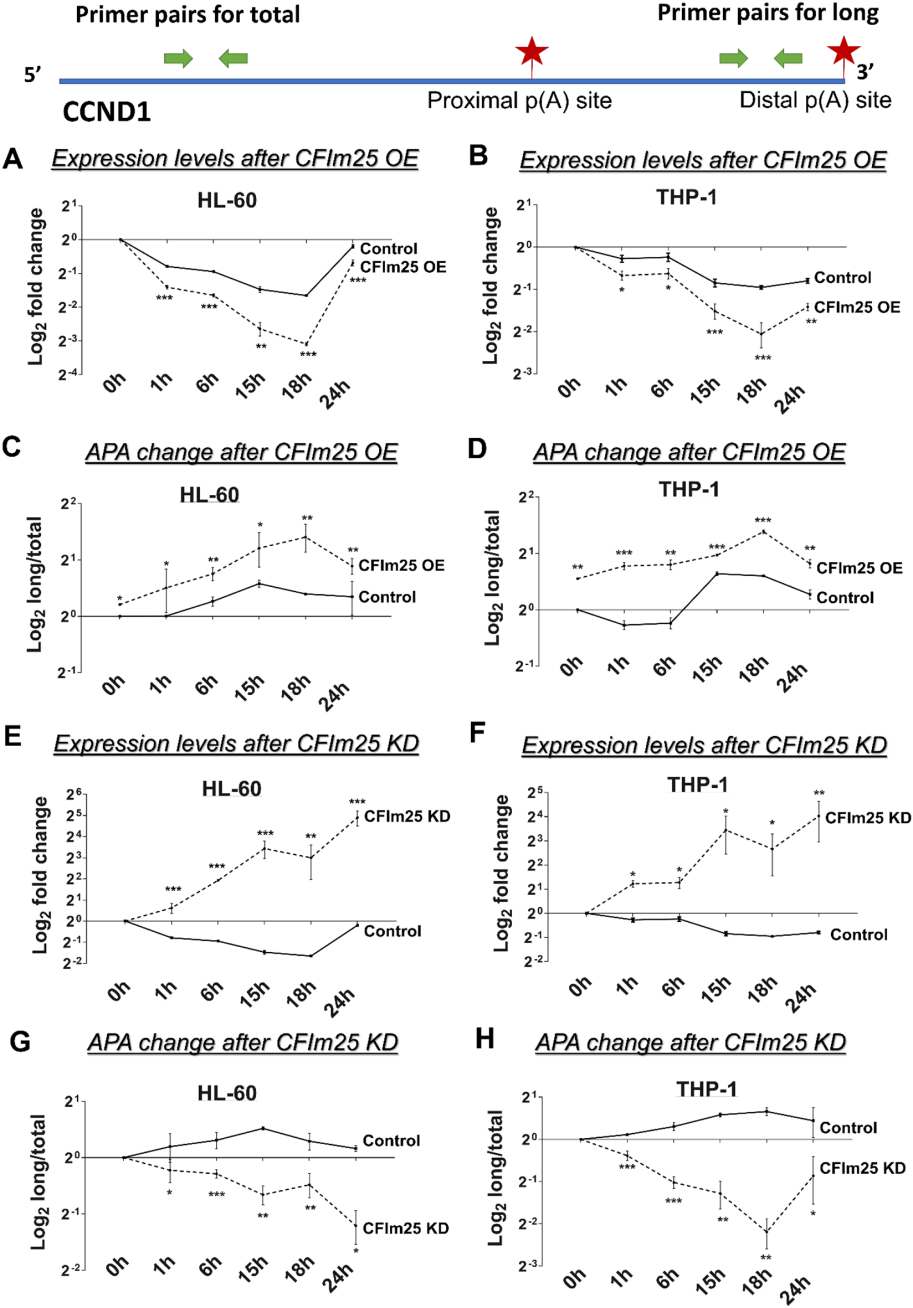
APA and expression of CCND1 after CFIm25 manipulation. Real-time quantitative PCR (RT-qPCR) analysis of the expression and distal PAS usage of CCND1 used two pairs of primers, where one represents the total transcript level and the other primer pair targets sequences just upstream of the distal PAS to detect long transcripts. Positions of primer pairs for each gene are provided in the top panel and sequences in Supplementary Table 2. (**A** and **B**) Fold changes (log_2_) in expression of total CCND1 mRNA during the 0–24 h time course post PMA treatment of HL-60 and THP-1 cells which are transfected with either control or constructs overexpressing CFIm25. Values are normalized to ACTB mRNA. (**C** and **D**) Changes in ratio of long to total transcript levels of control and CFIm25 overexpressing cells. (**E** and **F**) Expression changes after knocking down CFIm25 compared to controls. (**G** and **H**) Changes in ratio of long to total transcript levels of control and CFIm25-depleted cells. Graphs represent mean ± SE from three independent experiments. P value < 0.05 was considered significant, where * = *P* ≤ 0.05; **= *P* ≤ 0.01; *** = *P* ≤ 0.001

Usage of the proximal PAS to generate shorter isoforms of CCND1 (Cyclin D1) mRNA has been associated with increased cyclin D1 protein and increased cell proliferation of HEK293T, lung cancer, and breast cancer cells [19, 20, 49]. In addition, CFIm25 depletion in lung cancer cells decreased CCND1 distal PAS usage and enhanced cyclin D1 levels and cell proliferation [20]. Since our data showed that cyclin D1 protein levels were inversely correlated with CFIm25 levels in differentiating monocytes, we wanted to investigate whether the CCND1 APA status could contribute to these changes. We first determined the total level of CCND1 mRNA expression in HL-60 and THP-1 cell lines using primers designed to amplify the exon 2–3 junction (Fig. 4). CCND1 mRNA decreases in the control cells between 0 and 18 h and decreases even further upon CFIm25 overexpression (Fig. 4A and B). Next, we measured APA changes by a PCR-based method, where long isoforms were detected with a primer pair just upstream of the mapped distal PAS (Fig. 4), and the ratio of long/total mRNA expression was used to determine the change in usage of the distal site. Using this strategy, we found significantly increased usage of the distal sites, i.e., formation of longer isoforms upon CFIm25 overexpression during macrophage differentiation, along with an increase in the long mRNA isoforms (Fig. 4C and D and Supplementary Fig. 4A). In contrast, CFIm25 knockdown resulted in an increased expression of total mRNAs coupled with significant shortening of CCND1 transcripts during the course of differentiation (Fig. 4E-H and Supplementary Fig. 4B). Thus, the lengthening that we observe in CCND1 mRNAs could explain the decrease in cyclin D1 protein and contribute to the slowing of the cell cycle during macrophage differentiation.

### Global effect of CFIm25 overexpression on alternative polyadenylation during macrophage differentiation

To determine the effects of CFIm25 on other PAS usage during macrophage differentiation, we used the QuantSeq 3’ mRNA-sequencing method [50], which can provide information on differential APA events. PolyA+-selected RNA samples from HL-60 cells overexpressing CFIm25 and control cells after 6 h of PMA treatment were sequenced and analyzed to identify changes in isoform usage. We selected the 6 h timepoint because at this time, the control cells are entering a transition phase marked by slowing of the cell cycle and changes in the expression of the surface marker CD38 (Fig. 2C and E). We classified significant APA events as those where *P* ≤ 0.05 and found two genes to be lengthened (NFYC and UCK2) and eight genes to be shortened (CHMP4B, FERMT3, IREB2, OAZ1, SRI, TAB2, TBL1XR and TM9SF2). Detailed outputs for these APA events are supplied in Supplementary Table 3. Genome browser plots showing the positions and changes in PAS usage for these genes are shown in Supplementary Fig. 5.

**Fig. 5:**
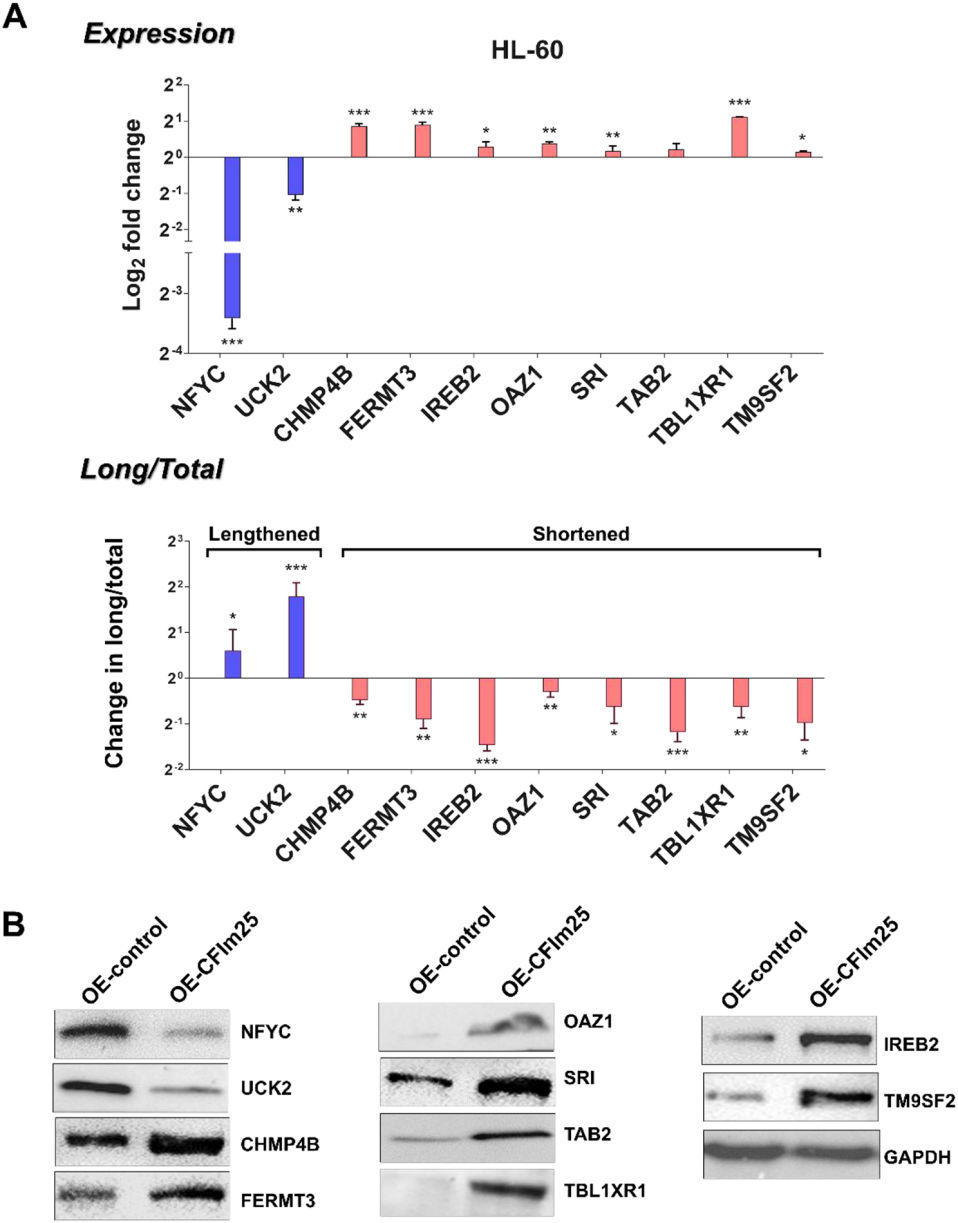
CFIm25 overexpression results in APA changes of specific genes and altered protein expression in PMA-treated HL-60 cells. (**A**) APA and expression analysis of CFIm25 target genes. Real-time quantitative PCR (RT-qPCR)–based analysis of the changes in expression (top panel) and distal PAS usage (bottom panel) of genes in HL-60 cells identified by QuantSeq as undergoing transcript lengthening (NFYC, UCK2) or shortening (CHMP4B, FERMT3, IREB2, OAZ1, SRI, TAB2, TBL1XR1 and TM9SF2) upon CFIm25 overexpression. Analysis was performed as described in Fig. 4. P value < 0.05 was considered significant, where * = *P* ≤ 0.05; **= *P* ≤ 0.01; *** = *P* ≤ 0.001. (**B**) Protein levels of genes with APA changes. Western blot analysis of proteins encoded by the shortened and lengthened genes in HL-60 cells overexpressing CFIm25 compared to control. GAPDH serves as the loading control

We used the PCR-based method [10, 23] to confirm a change in mRNA isoforms from the genes that differed in their 3’ ends. Using a coding sequence primer pair (Supplementary Table 2), we found that while total mRNAs of the lengthened NFYC and UCK2 genes in HL-60 cells were decreased, TAB2 mRNAs stayed the same and all others (CHMP4B, FERMT3, IREB2, OAZ1, SRI, TBL1XR1 and TM9SF2) were increased upon CFIm25 overexpression (Fig. 5A, top panel). Transcripts extending beyond the proximal PASs were detected with a primer pair just upstream of mapped distal PASs (Supplementary Table 2), and the ratio of long isoform/total transcripts was used to determine change in usage relative to the control cells (Fig. 5A, bottom panel). Using this strategy, we found an increased use of distal p(A) sites of NFYC and UCK2 mRNAs (lengthening) and a decreased usage (shortening) for the other genes after CFIm25 overexpression (Fig. 5A, bottom panel). These results are in accordance with the QuantSeq data (Supplementary Fig. 5). Furthermore, we observed similar directional changes in protein levels as seen with the mRNA expression (Fig. 5B), in agreement with studies suggesting that shortened 3′ UTRs often lead to an increased stability of the mRNAs and an increased protein output [3–5]. We found similar changes in mRNA expression, PAS usage, and protein levels for these genes upon CFIm25 overexpression in THP-1 cells, except that SRI transcripts were not shortened (Supplementary Fig. 6A and 6B). The possible roles of the genes undergoing CFIm25-induced APA are described later in the Discussion.

### CFIm25 affects the alternative polyadenylation of effectors of the NF-κB pathway

**Fig. 6:**
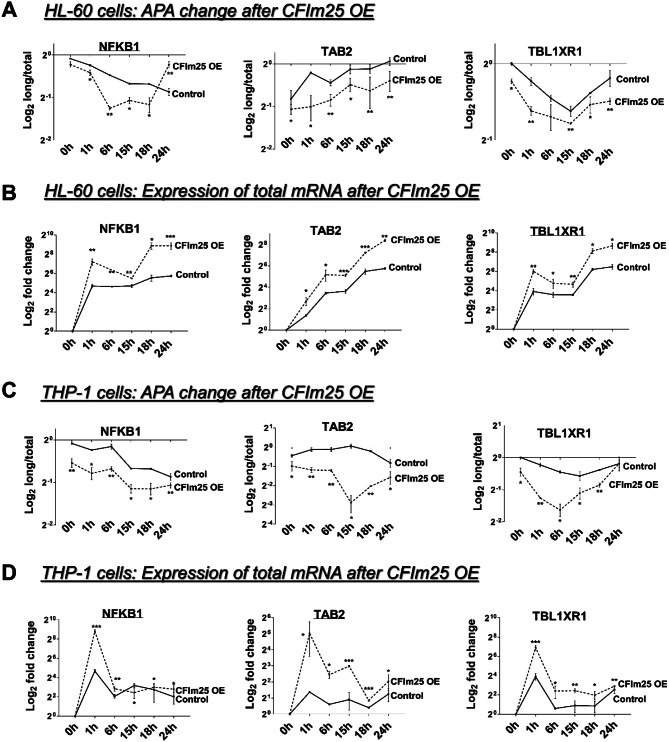
CFIm25 overexpression affects mRNA levels and APA of effectors of the NF-κB pathway. Real-time quantitative PCR (RT-qPCR)–based analysis of the change in distal PAS usage (the long/total ratio) and the expression of total NFKB1, TAB2 and TBL1XR1 mRNAs after overexpression of CFIm25 and treatment with PMA for 0–24 h in HL-60 (**A**, **B**) and THP-1 (**C**, **D**) cells. The analysis was performed as described in Fig. 4

NF-κB has been implicated as a master regulator of monocytes and macrophages [24], and activation of this pathway could be beneficial for acute myeloid leukemia (AML) patients by inducing terminal myeloid differentiation [51]. Of the targets we examined that undergo alterations in their 3’ UTR lengths, TAB2 and TBL1XR1 directly contribute to the NF-κB pathway [28–31, 52]. NFKB1, encoding the p50 subunit of NF-κB, is also reported to exhibit APA site switching during the antiviral immune response [53]. While the two cell lines showed differences in the timing or magnitude of changes in mRNA during the differentiation time course in response to CFIm25 overexpression, both HL-60 and THP-1 cells showed consistent shortening of the 3’ UTRs of NFKB1, TAB2 and TBL1XR1 mRNAs (Fig. 6A and C) that was coupled with increased levels of total mRNA and a decrease in long mRNA isoforms (Fig. 6B and D and Supplementary Fig. 7A and 7B). In contrast, depletion of CFIm25 caused an increase in the long mRNA isoforms and the long/total ratio, and a decrease in the amount of total mRNA (Supplementary Fig. 8).

We also analyzed APA changes in the 3’ UTRs of NFKB1, TAB2 and TBL1XR1 mRNAs in primary human blood monocytes differentiated into macrophages with M-CSF for seven days compared to those without M-CSF exposure. Seven days of M-CSF treatment causes a large increase in CFIm25 protein (Supplementary Fig. 1G). Our RNA analysis shows that the 3’UTRs of all three mRNAs are indeed significantly shortened during differentiation, as observed by RT-qPCR analysis (Supplementary Fig. 7C). The shifts in APA of these mRNAs concordant with the increase in CFIm25 expression suggests that CFIm25 will have a similar regulatory role in the transition of primary monocytes to macrophages.

### CFIm25 overexpression increases the protein levels of the APA-regulated NF-κB effector genes and that of NF-κB targets

**Fig. 7:**
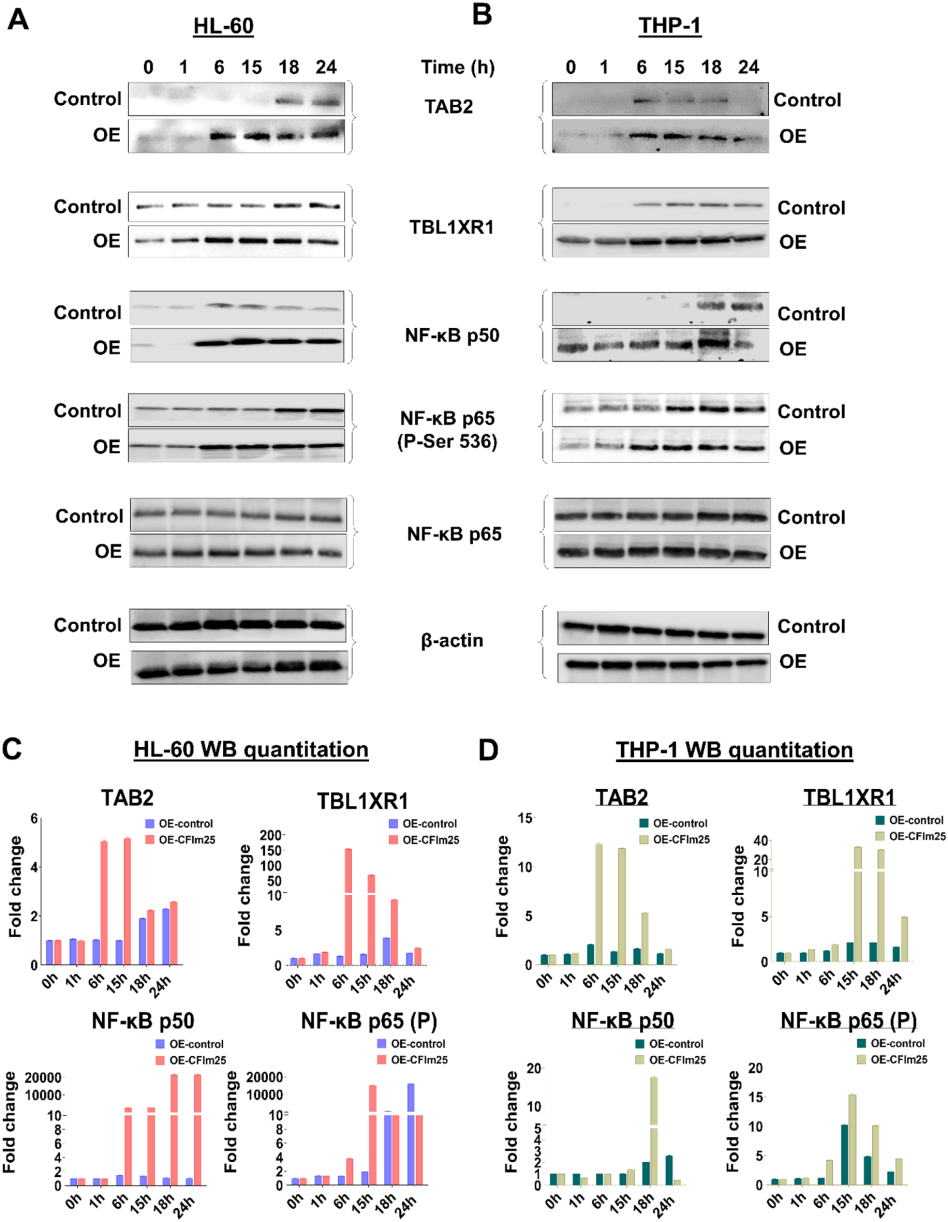
CFIm25 overexpression alters levels of the effectors of the NF-κB pathway. (**A** and **B**) Western blot analysis the indicated proteins during the differentiation time course for HL-60 (**A**) and THP-1 (**B**) cells with and without overexpression of CFIm25 (0–24 h). (**C** and **D**) Quantitation of the changes in Western blot signal expressed as fold-changes. b-actin serves as a loading control

**Fig. 8:**
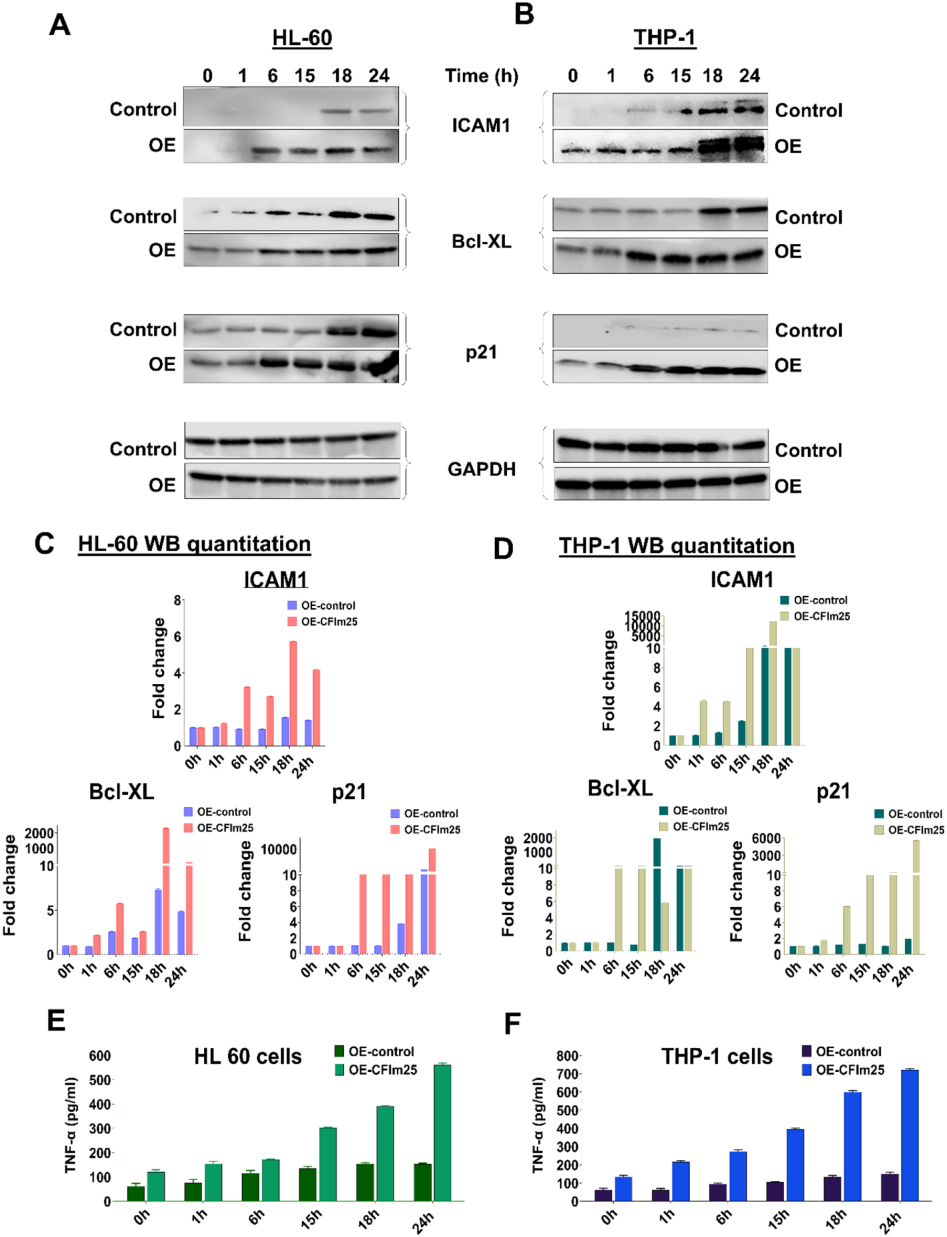
CFIm25 overexpression affects the targets of the NF-κB pathway. (**A** and **B**) Western blot analysis the indicated proteins during the differentiation time course for HL-60 (**A**) and THP-1 (**B**) cells with and without overexpression of CFIm25 (0–24 h). (**C** and **D**) Quantitation of the changes in Western blot signal expressed as fold-changes. GAPDH serves as a loading control. (**E** and **F**) ELISA of TNF-α in HL-60 (**E**) and THP-1 (**F**) cell supernatants upon overexpression of CFIm25 with respect to controls after induction of differentiation

The mRNA changes seen with CFIm25 overexpression were associated with changes in the levels of NFKB1, TAB2 and TBL1XR1 proteins, with more and earlier protein expression observed over the 24-hour differentiation time course (Fig. 7). We also examined the expression of the NF-κB p65 (RelA) subunit and downstream targets by western blot. An earlier increase in the levels of phosphorylated p65 after CFIm25 overexpression indicates that the NF-κB pathway is hyperactivated (Fig. 7). Important downstream targets of the NF-κB pathway [54], such as the Cyclin Dependent Kinase inhibitor p21, the adhesion molecule ICAM1, and the anti-apoptotic factor Bcl-XL exhibited greater and earlier increases in protein levels in both cell lines upon CFIm25 overexpression (Fig. 8A-D). As it is well known that NF-κB mediates the induction of cytokines in monocytes and macrophages [24], we also used ELISA to measure the levels of TNF-α, which facilitates communication between immune cells and other cell types in the body to coordinate responses to infection or injury. CFIm25 overexpression caused a strong increase in TNF-α in both HL-60 and THP-1 cells (Fig. 8E and F).

### Macrophage phenotypes in CFIm25-overexpressing cells rely on the NF-κB pathway

**Fig. 9:**
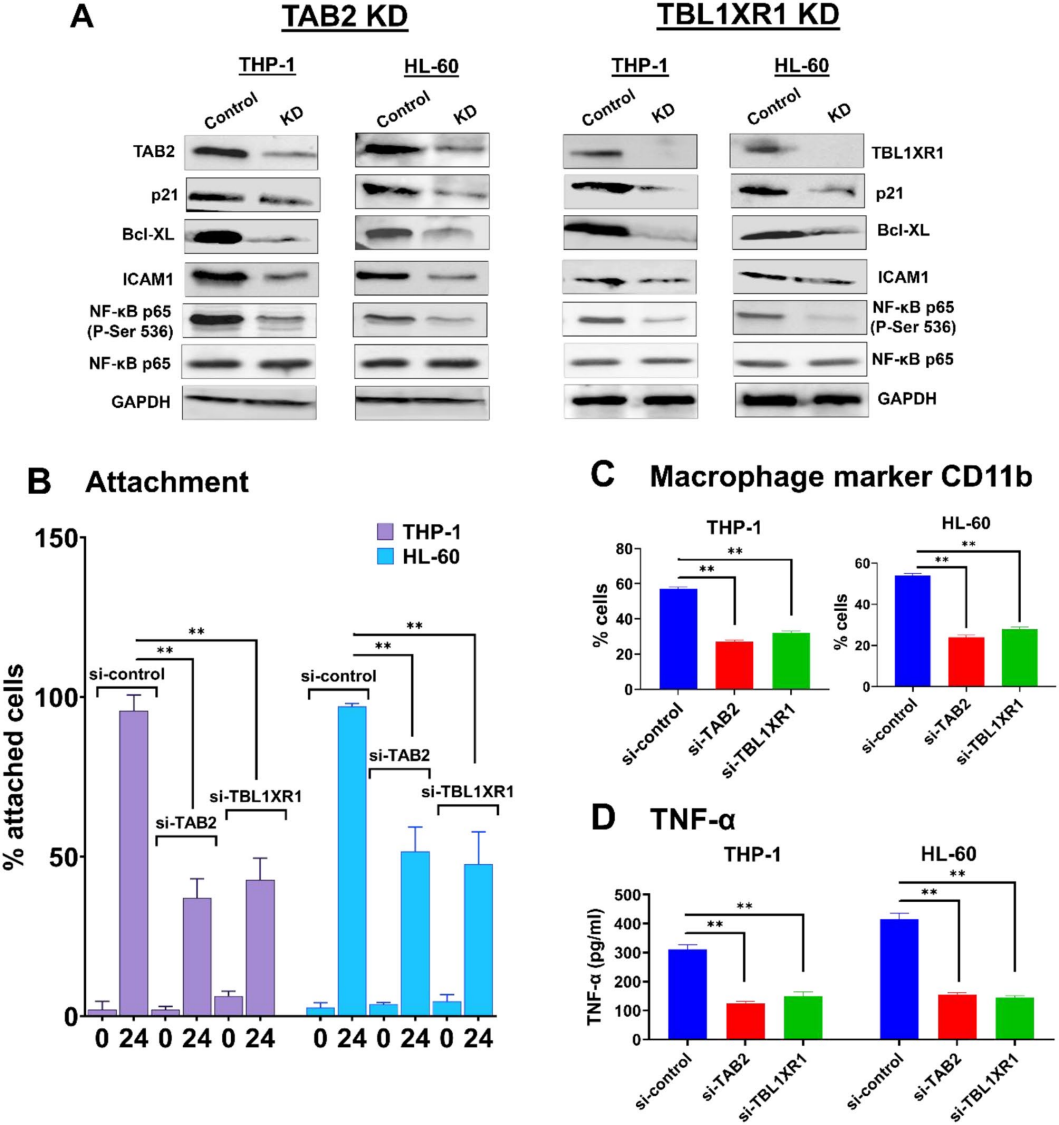
TAB2 and TBL1XR1 mediate macrophage phenotypes in CFIm25-overexpressing cells. (**A**) Western blots confirming TAB2 and TBL1XR1 knockdown and showing effects on NF-κB p65 and NF-κB targets. Cells overexpressing CFIm25 were treated with control siRNAs or siRNA against TAB2 or TBL1XR1 and induced to differentiate with PMA for 0–24 h (**B**) Effects on cell attachment, assayed as described for Fig. 1. (**C**) TAB2 and TBL1XR1 knockdowns suppress CD11b levels as assayed by flow cytometry in THP-1 or HL-60. (**D**) TAB2 and TBL1XR1 knockdowns suppress TNF-a levels as assayed by ELISA. P value < 0.05 was considered significant, where * = *P* ≤ 0.05; **= *P* ≤ 0.01

In the previous sections, we showed that increasing the level of CFIm25 led to faster differentiation and caused shortening of the mRNAs and greater protein expression of TAB2 and TBL1XR1, both positive effectors of the NF-κB pathway. To confirm the role of TAB2 and TBL1XR1, we performed siRNA-mediated knockdown of these genes in THP-1 and HL-60 cells overexpressing CFIm25 and induced them to differentiate with PMA. The knockdown was confirmed by Western blot (Fig. 9A). Depletion of either TAB2 or TBL1XR1 decreased cell attachment and expression of surface-level CD11b (Fig. 9B and C, and Supplementary Fig. 9), indicating that these NF-κB regulators were important for macrophage differentiation. Depletion of either TAB2 or TBL1XR1 in both THP-1 and HL-60 cells also suppressed accumulation of phosphorylated NF-κB p65 and production of targets of NF-κB such as p21, Bcl-XL, ICAM1, and TNF-a (Fig. 9A and D). These findings further strengthen the mechanistic connection between CFIm25, its target transcripts, and NF-κB pathway activation.

**Fig. 10:**
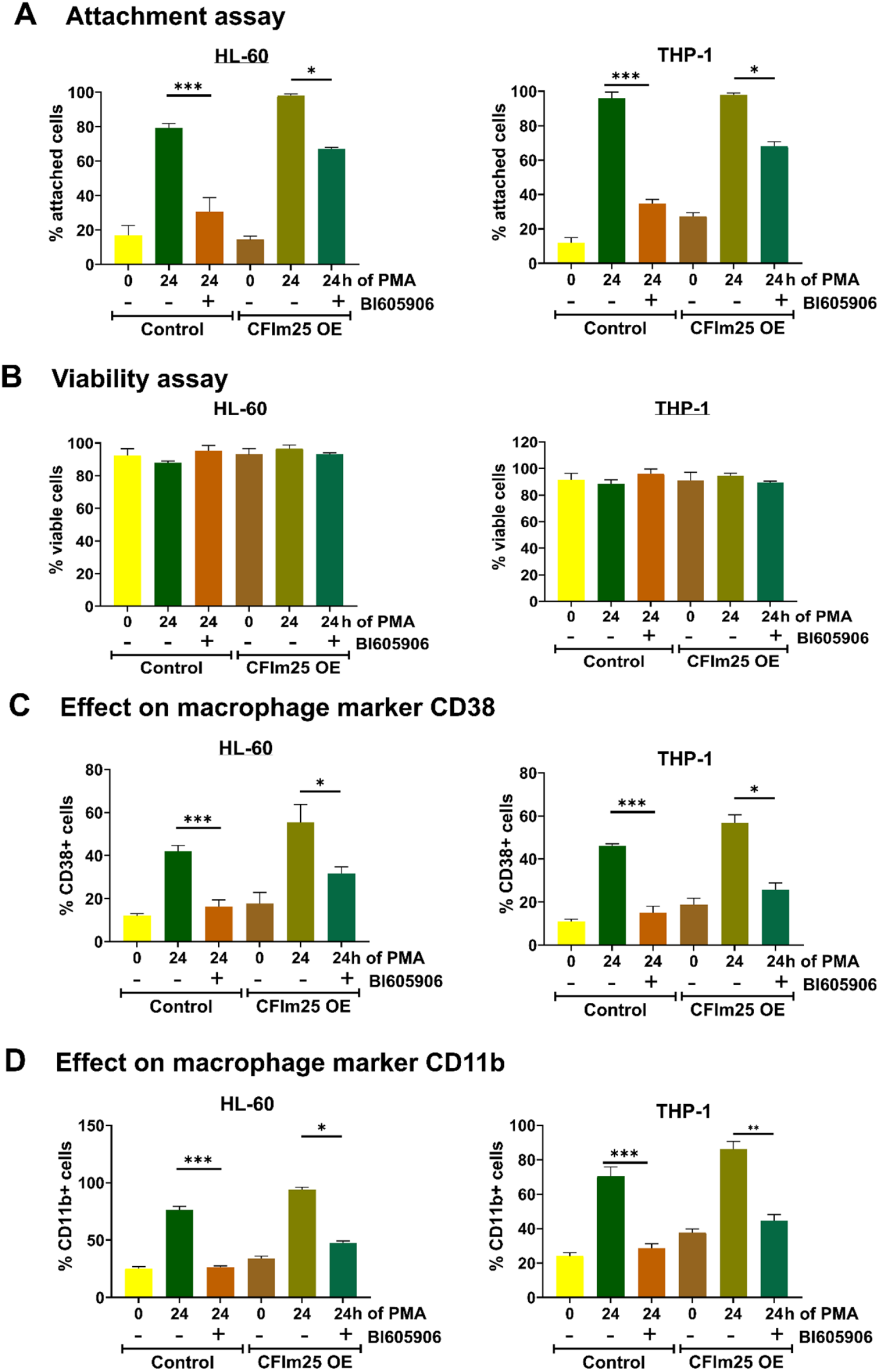
CFIm25 overexpression provides resistance to the NF-κB inhibitor BI605906 (**A**) Attachment assay. HL-60 and THP-1 control cells or cells overexpressing CFIm25 were incubated with or without BI605906 and then treated with PMA for 0 and 24 h, and the percentage of cells attached at each time point determined. (**B**) Viability assay. The viability of cells was determined by the resazurin conversion assay. (**C** & **D**) Effect on macrophage markers CD38 and CD11b by flow cytometry of HL-60 and THP-1 control cells or those overexpressing CFIm25 with or without treatment with NF-κB inhibitor. The expression of CD38 and CD11b is shown as a percentage of CD38 + or CD11b + cells. Data is representative of at least three biological replicates and is plotted as mean ± SE. P value < 0.05 was considered significant, where * = *P* ≤ 0.05; *** = *P* ≤ 0.001

To further assess NF-κB activation, we treated control or CFIm25 overexpressing HL-60 or THP-1 cells with the NF-κB pathway inhibitors BI605906 or BAY11-7082 and subjected them to attachment, viability and flow cytometric analysis for macrophage markers. BI605906 specifically inhibits the IKKβ subunit of IKK, the kinase that phosphorylates and induces degradation of IκB, a protein which sequesters NF-κB in the cytoplasm [55]. BAY 11-7082 is also commonly used as an NF-κB inhibitor but works indirectly by inhibiting E2 ubiquitin ligases that activate IKK, and it could also affect other pathways [51, 56]. Treatment with BAY 11-7082 and importantly, BI605906, greatly reduced the attachment of the PMA-treated control HL-60 and THP-1 cells without affecting their viability (Fig. 10A and B and Supplementary Figs. 10–11) and decreased the percentage of cells expressing the macrophage markers CD38 and CD11b (Fig. 10C and D and Supplementary Figs. 10–11, indicating impeded transition of monocytes to macrophages. In contrast, cells overexpressing CFIm25 showed less reduction in attachment and marker expression after BI605906 or BAY 11-7082 treatment compared to the control cells treated with the inhibitors (Fig. 10C and D and Supplementary Figs. 10–11). These results indicate that overexpression of CFIm25 provides resistance to chemical inhibition of the NF-κB pathway. While it is possible that the effects of CFIm25 on other pathways counteract the negative effects of the NF-κB inhibitors, the hyperactivation of NF-κB signaling by CFIm25 that we have documented probably contributes to the resistance.

## Discussion

**Fig. 11:**
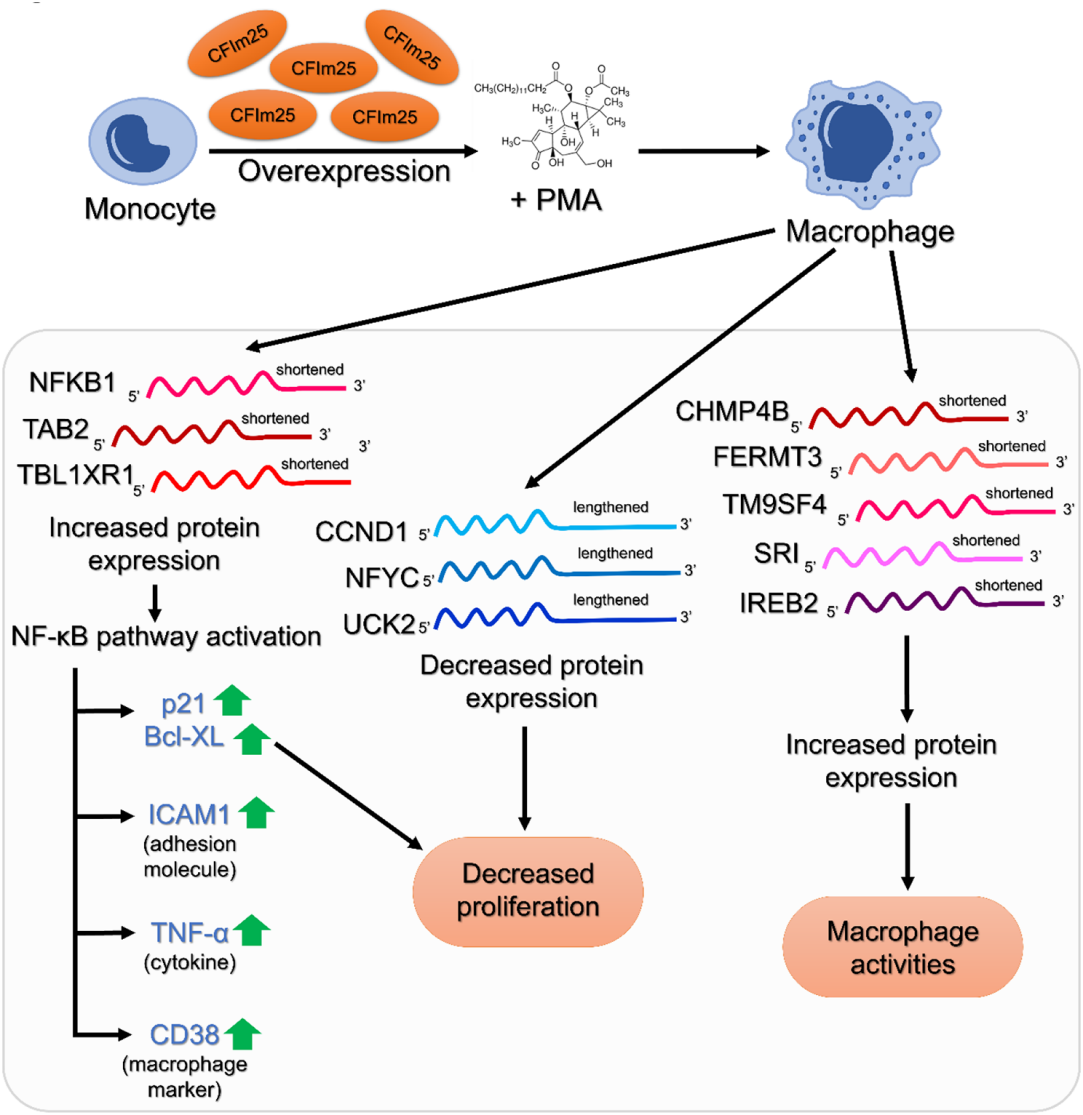
Summary of findings and a model to explain CFIm25-mediated regulation. Increased expression of CFIm25 in monocytic cells leads to more efficient differentiation to macrophages through regulated APA of critical genes and activation of the NF-κB pathway and its target genes

Monocyte differentiation yields macrophages primed to respond to stimuli through innate immune functions, such as inflammation or tissue repair. Our study aimed to deepen our currently rudimentary understanding of how alternative polyadenylation (APA) of mRNA contributes to macrophage differentiation. Previously, we observed that CFIm25, a protein known for its role in APA regulation in other contexts, increases during macrophage differentiation of monocytic cell lines [6]. In this study, we demonstrate that the increase in CFIm25 has functional significance, promoting cell cycle arrest, the adherence necessary for migration, and expression of macrophage markers CD38 and CD11b. We also show that CFIm25 impacts macrophage differentiation by activating the NF-κB pathway (Fig. 11). While other signaling pathways are likely involved, we focused on NF-κB because of its well-documented activity in macrophage differentiation and function. Moreover, our differentiation inducer, PMA, is known to activate it [24, 57, 58]. Our findings are particularly novel because they show that CFIm25 not only affects expression of genes needed for macrophage activities but also fine-tunes the NF-κB pathway to drive differentiation. CFIm25 overexpression enhanced several outputs of the NF-κB pathway [27, 54], including the Cyclin Dependent Kinase inhibitor p21, the anti-apoptotic and anti-proliferative protein Bcl-XL, the adhesion molecule ICAM1, and the cytokine TNF-α. The increased phosphorylation at Ser 536 of the NF-κB p65 subunit and higher levels of the p50 subunit further indicate enhanced activation. Additionally, CFIm25-induced TNF-α may contribute to the increased CD38 production [59, 60]. Importantly, the effects of CFIm25 overexpression on differentiation were dampened by depletion of the positive NF-κB effectors TAB2 and TBL1XR1, and CFIm25 overexpression lessened the repressive effects of NF-κB inhibitors, supporting a pivotal role for CFIm25 in NF-κB regulation.

Our results suggest that APA of key NF-κB effectors contributes to pathway activation. When CFIm25 is overexpressed, the 3’ UTRs of TAB2, TBL1XR1, and NFKB1 are shortened, and we observe increased mRNA and protein levels. These findings align with those of Jia et al. [53], who documented similar shortening of NFKB1 mRNAs in virus-infected macrophages and showed that a luciferase reporter with the short NFKB1 3’ UTR made more protein than one with the long 3’ UTR. NFKB1 encodes the precursor of the NF-κB p50 subunit [61], while TAB2 is part of the TAK1 kinase complex that drives macrophage differentiation and activates NF-κB in response to inflammatory signals [28, 62, 63]. TBL1XR1 binds to p65 and facilitates its nuclear translocation, leading to transcription of NF-κB target genes [29–31, 64]. By promoting the APA and protein output of these mRNAs, CFIm25 overexpression can enhance NF-κB activity and drive the monocyte-to-macrophage transition.

Interestingly, the CFIm25-mediated increases in protein levels do not always correspond directly with mRNA levels. For example, in HL-60 cells overexpressing CFIm25 (Figs. 6 and 7), the mRNAs for TAB2 and TBL1XR1 are shortened and increase in levels after 1 h of PMA treatment, but a CFIm25-dependent protein increase is not seen until 6 h. This lag suggests additional regulatory mechanisms, perhaps at the levels of transcription, translation, or protein stability, are likely at play to fine-tune protein levels. While we do not know what these underlying mechanisms might be, our study documents that CFIm25 overexpression increases the protein expression of the NF-κB effectors p50, TAB2, and TBL1XR1 during macrophage differentiation. Additionally, CFIm25 likely influences some of the other signaling pathways, such as PI3K/AKT, MAPK, JAK-STAT, TGF-β, Wnt, Notch, and Hedgehog, which coordinate with NF-κB in macrophages. For example, TAB2 also activates MAPKs, which are essential for macrophage differentiation [28, 65–71], and CFIm25-mediated APA might modulate crosstalk between these pathways.

While NF-κB is primarily known for its role in inflammation [24, 54], it can also induce cell cycle arrest at the G1/S transition in several cell types [72–75], and slowing of proliferation is a key event in monocyte-to-macrophage differentiation [33]. CFIm25 overexpression caused cell cycle slowing to occur earlier during differentiation, while its depletion blocked it. Our findings indicate that CFIm25 slows the G1 to S phase at least in part by increasing levels of the NF-κB targets p21 and Bcl-XL, essential regulators of cell cycle progression. CFIm25 also affects the cell cycle independently of NF-κB. For instance, it binds UGUA elements in the 3’ UTR of CCND1 mRNA, promoting distal PAS usage and reducing Cyclin D1 protein levels, which slows the G1-S transition. We observed 3’ end lengthening of CCND1 and Cyclin D1 depletion in differentiating monocytes, which was further enhanced by CFIm25 overexpression. In addition, CFIm25-induced 3’ UTR lengthening correlates with reduction of mRNA and protein levels of NFYC and UCK2, both of which promote cell cycle progression, while 3’ UTR shortening is associated with increased OAZ1, which inhibits proliferation and promotes differentiation [76–79]. These findings suggest that CFIm25 plays a multifaceted role in regulating cell cycle progression during macrophage differentiation. CFIm25-mediated APA of transcripts without a known relationship to NF-κB, such as CHMP4B, FERMT3, SRI, TM9SF4, and IREB2, likely impacts other key macrophage functions (Fig. 11). These genes show shortened 3’ UTRs and increased expression with CFIm25 overexpression. CHMP4B, part of the ESCRT-III complex, is involved in phagosome maturation and antigen presentation [80]. FERMT3 and TM9SF4 play roles in macrophage adhesion and phagocytosis [81, 82]. SRI may influence macrophage migration, as it does in cancer cells [83]. IREB2 is essential for macrophage iron recycling [84]. Together, these APA events highlight a broader role of CFIm25 in promoting macrophage functionality.

Our study provides a foundation for several intriguing avenues of further investigation:

1) We do not know what regulates the increased expression of CFIm25 during macrophage differentiation, but it could involve multiple mechanisms such as changes in mRNA processing, translation, or protein stability. In line with this possibility, CFIm25 mRNAs have been reported to use alternative 3’ UTR PASs and to be regulated by miRNAs in other cell types [13, 85–87] and CFIm25 protein stability is decreased in non-small cell lung cancer [88]. This suggests that similar mechanisms might be involved in the context of macrophage differentiation.

2) Our 3’ end focused sequencing only identified ten genes with significant APA after CFIm25 overexpression, possibly due to a dose effect in which the level of CFIm25 overexpression in our experiments may only impact APA in a subset of transcripts, and because we applied very stringent thresholds to identify genes with the most significant APA. Despite the small number, all identified genes had links to macrophage differentiation and function. It is likely that APA affects other genes important for macrophage differentiation, but these events may be regulated by other RNA binding proteins or be manifested at a different point in the differentiation timeline than the one we examined. Identification of these genes and the consequences of their APA will be important to fully understand the impact of APA during macrophage differentiation.

3) In addition to polyadenylation, CFIm25 has been reported to affect processes such as alternative splicing, circular RNA formation, and RNA polymerase II occupancy in the body of transcribed genes [14, 89]. However, the impact of these activities of CFIm25 on macrophage differentiation remains unknown.

## Conclusion

Our study demonstrates that CFIm25 expedites the monocyte-to-macrophage transition and regulates the APA of key genes and their respective proteins, including ones involved in cell cycle control and NF-κB signaling. Our findings reveal a novel role for CFIm25 in immune cell differentiation and provide insights into how therapeutic manipulation of APA could better promote the resolution of infections, cancer, and other diseases affected by macrophage activity.

## Electronic supplementary material

Below is the link to the electronic supplementary material.

Supplementary Material 1

## Data Availability

The datasets generated during and/or analyzed during the current study are available in the GEO repository with accession number GSE254061.

## Abbreviations

C/P: Cleavage and polyadenylation
PMA: Phorbol Myristate Acetate
APA: Alternative polyadenylation
PAS: Poly(A) Site
