# Supplementary material for: The alternative polyadenylation regulator CFIm25 promotes macrophage differentiation and activates the NF-κB pathway
Source: Cell Commun Signal. 2025 Feb 28;23:115. doi: 10.1186/s12964-025-02114-1 (PMC11871739; doi:10.1186/s12964-025-02114-1)

Supplementary Material

1 Supplementary Tables and Figures

1.1 Supplementary Tables

Supplementary Table 1. Antibodies used in this study:

Western blot antibodies:

Catalogue no.	Name	Host	MW	Vendors
SC-81109	CFIm25	Mouse monoclonal	26 kDa	Santa Cruz Biotechnology
SC-374650	CD38	Mouse monoclonal	45 kDa	Santa Cruz Biotechnology
SC-8396	Cyclin D1	Mouse monoclonal	37 kDa	Santa Cruz Biotechnology
SC- 56	PCNA	Mouse monoclonal	36 kDa	Santa Cruz Biotechnology
SC-47778	β-actin	Mouse monoclonal	43 kDa	Santa Cruz Biotechnology
SC-365062	GAPDH	Mouse monoclonal	37 kDa	Santa Cruz Biotechnology
SC-374015	Lamin B1	Mouse monoclonal	67 kDa	Santa Cruz Biotechnology
3033S	Phospho (Ser 536) NF-κB-p65	Rabbit monoclonal	65 kDa	Cell Signaling Technologies
8242S	NF-κB-p65	Rabbit monoclonal	65 kDa	Cell Signaling Technologies
SC-8414	NF-κB-p50	Mouse monoclonal	50 kDa	Santa Cruz Biotechnology
SC-8439	ICAM1	Mouse monoclonal	85-110 kDa	Santa Cruz Biotechnology
SC-6246	p21	Mouse monoclonal	21 kDa	Santa Cruz Biotechnology
SC-8392	Bcl-XL	Mouse monoclonal	30 kDa	Santa Cruz Biotechnology
SC-398188	TAB2	Mouse monoclonal	83 kDa	Santa Cruz Biotechnology
SC-100908	TBL1XR1	Mouse monoclonal	55 kDa	Santa Cruz Biotechnology
28329-1-AP	IREB2	Rabbit polyclonal	95-110 kDa	Proteintech
Ab-7839	FERMT3	Rabbit polyclonal	76 kDa	Abclonal Antibody
136183-1-AP	CHMP4B	Rabbit polyclonal	25-33 kDa	Proteintech
25595-1-AP	TM9SF2	Rabbit polyclonal	70 kDa	Proteintech
SC-100859	SORCIN	Mouse monoclonal	22 kDa	Santa Cruz Biotechnology
A7444	OAZ1	Rabbit polyclonal	25 kDa	Abclonal Antibody
SC-390985	NFYC	Mouse monoclonal	40 kDa	Santa Cruz Biotechnology
422301	Human TruStain FcX™	Monoclonal Antibody	F_c blocker	Biolegend

Flow cytometry antibody:

Catalogue no.	Name	Host	Fluorophore
Biolegend 102707	CD38	Mouse	PE
Proteintech PE-65116	CD11b	Mouse	PE

Supplementary Table 2. Primers used in this study:

Purpose	Primer name	Forward primer 5'-3'	Reverse primer 5'-3'
RT-qPCR for gene expression	ACTB total	CATGTACGTTGCTATCCAGGC	CTCCTTAATGTCACGCACGAT
	CCND1 total	AACTACCTGGACCGCTTCCT	CCACTTGAGCTTGTTCACCA
	NFYC total	GGACTCCTGAGCAGAGTTGT	GCAAAGAGTACAGGCGCTTC
	UCK2 total	CGGAAAGCGGAGGGAGTC	CTATAAGGAAGGGCTCGCC
	CHMP4B total	GGTGTTCGGGAAGCTGTTC	TTTTGGTGCCGTGCTTCTTG
	FERMT3 total	AGAGAAAGGAGGGCAGGAAG	TCCTCCTCTCCCACAAACAC
	IREB2 total	AGCCTGCTTCCTTCTTTCCT	CCATCGCCGGAGACCATATT
	OAZ1 total	GCATCTATAAAGGCGGGCG	CCTTCCTTCTCTCTGGCGAA
	SRI total	CTCAGGATCCGCTGTATGGT	TGTATCCTCCAGCAATGCCA
	TAB2 total	AGGGAGGGCTGAGGTGTC	CACTAGGGCAGCCGTCTC
	TBL1XR1 total	CTGTCCCTGATAGATGCCGT	TTCTCCTCCCCATTTGCTGT
	TM9SF2 total	CAACTATCATGAGCGCGAGG	CTTGCACTCGTCGCTCTTTT
	NFKB1 total	CAAGCAGCTCTGCAGCAG	ACTGTCATAGATGGCGTCTG
RT-qPCR of long transcript for APA analysis	CCND1 long	ACGCTTTGTCTGTCGTGATG	GTGCAACCAGAAATGCACAG
	NFYC long	CCAAGACTTGCCACGTTGTT	GGCAATGAATCCACCCACTC
	UCK2 long	GCCTCTCACTCCTTCACACT	CAAGGTGAACTGGAGACCCT
	CHMP4B long	CTTGCCGCACATCTCTTTGT	AAACTCCAGAAGACCAGGGG
	FERMT3 long	GGCCAGACGCTGTACC	CAAGAAAGAACTCGTTTGAAAC
	IREB2 long	GGTAACAAGGTCGTGTGCAT	AAGCCAAAGTCCACCCTCTT
	OAZ1 long	GTGTTTGTGATACTGAAGTATTTGC	CAAGTTAAAAGACTAAGACTGTTTCC
	SRI long	TGAGCCATTATCAGTCATGCC	ACCCAAGTGCGTCTATGTCA
	TAB2 long	AGACCCAAAGCCCTTACGTT	ACCCAGCTAAATCACAAAACCA
	TBL1XR1 long	TAAACCAGCCCATGACAGGT	AGGGGAACTGCAACAACAAC
	TM9SF2 long	CCTGGACATTAGCAATCACTAGC	CAGCAAGCAGAGAGACCCTA
	NFKB1 long	CGTTCCTATTGTCATTAAAGGTATC	ATGGCACATCAAGTGACTCTC

Supplementary Table 3. Outputs for APA events identified by QuantSeq 3' mRNA **sequencing.** Table outlining the genes undergoing shortening or lengthening upon CFIm25 overexpression. In this table, chr: chromosome number where the gene is located, strand: the strand of chromosome on which the gene is coded, Prx_pA_pos: position of the proximal poly(A) site, Dis_pA pos: position of the distal poly(A) site, RED: relative expression difference (RED) between proximal and distal p(A) isoforms in overexpression vs. control samples (RED = difference in log_2(ratio) of read numbers of two p(A) isoforms between two samples), pval.fisher.adj: p value calculated for the RED score by Fisher's exact test and change: whether the gene is significantly shortened or lengthened.

Symbol	Description	chr	Strand	Prx_pA _pos	Dis_pA pos	RED	pval. fisher. adj	Change
**NFYC**	nuclear transcription factor Y subunit gamma	chr 1	+	41,236,778	41,237,273	1.39	2.07e-02	lengthened
**UCK2**	uridine-cytidine kinase 2	chr 1	+	165,877,345	165,880,855	0.99	1.99e-02	lengthened
**CHMP4B**	charged multivesicular body protein 4B	chr 20	+	32,441,636	32,442,169	-0.82	1.36e-02	shortened
**FERMT3**	FERM domain containing kindlin 3	chr 11	+	63,991,299	63,991,363	-0.95	2.49e-02	shortened
**IREB2**	iron responsive element binding protein 2	chr 15	+	78,792,879	78,793,794	-0.63	2.54e-02	shortened
**OAZ1**	ornithine decarboxylase antizyme 1	chr 19	+	2,273,238	2,273,323	-0.87	1.36e-02	shortened
**SRI**	sorcin	chr 7	-	87,835,606	87,834,431	-1.1	1.01e-02	shortened
**TAB2**	TGF-beta activated kinase 1 (MAP3K7) binding protein 2	chr 6	+	149,732,659	149,732,741	-1.26	2.90e-02	shortened
**TBL1XR1**	TBL1X/Y related 1	chr 3	-	176,741,135	176,738,543	-0.85	3.75e-02	shortened
**TM9SF2**	transmembrane 9 superfamily member 2	chr 13	+	100,215,064	100,215,643	-1.01	1.01e-02	shortened

Supplementary Figures.

Supplementary Figure 1. Flow cytometry analysis of HL-60 and THP-1 cells treated with PMA and analysis of CFIm25 protein levels during primary monocyte differentiation. (A and B) Representative raw data for cell cycle analysis of HL-60 **(A)** and THP-1 **(B)** cells treated with PMA for the indicated hours. Cell cycle was studied using propidium iodide (PI) staining of DNA, followed by flow cytometry, with 10,000 events acquired for each sample. A representative histogram of DNA content (X-axis, PI fluorescence) versus cell counts (Y-axis) is displayed, and peaks for G0/G1 (purple), S (yellow), and G2/M (green) indicated. **(C and D)** Representative dot plots for flow cytometry of CD38 at different times during differentiation of HL-60 **(C)** and THP-1 **(D)** cells, where the percentage on the Y axis represents cells positive for CD38, with FSC on the X axis. **(E)** Morphological changes after treating human primary monocytes with M-CSF for 5 and 7 days. **(F)** Western blot showing expression of the indicated macrophage marker proteins after M-CSF treatment of primary monocytes, with GAPDH as the loading control. **(G)** Western blot showing expression of CFIm25 after M-CSF treatment of primary monocytes, with β-actin as the loading control.

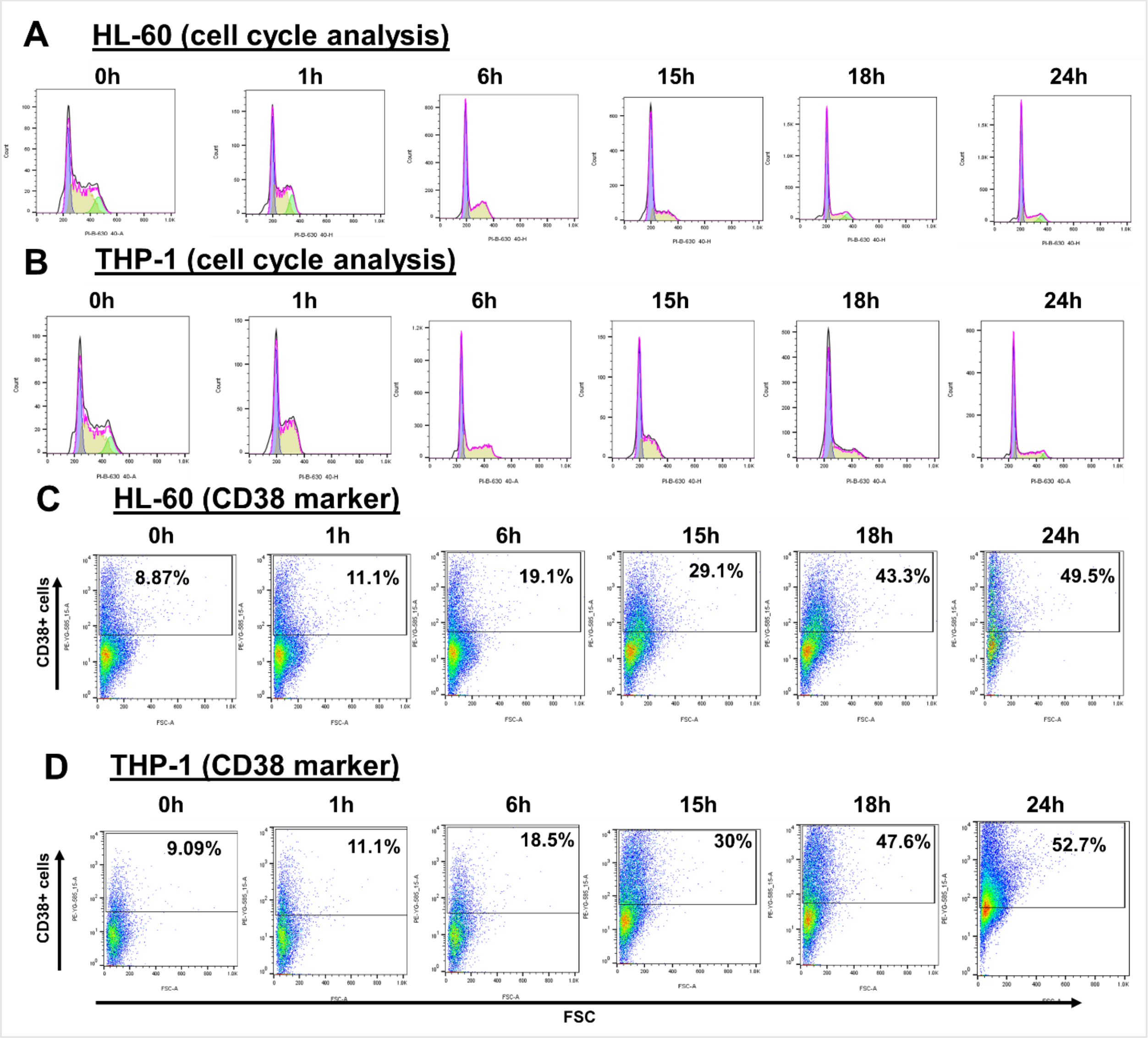

Supplementary Figure 1E-1G

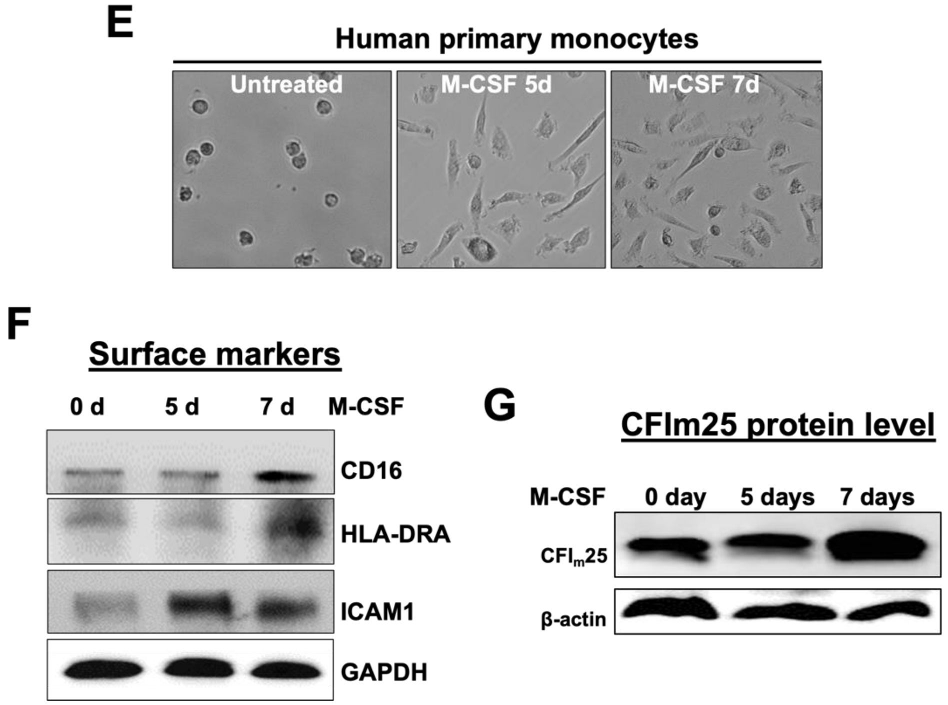

Supplementary Fig 2. Effects of CFIm25 overexpression. (A and B) Attachment and viability assays in (A) HL-60 and (B) THP-1 cells overexpressing CFIm25. For the attachment assay (*left*), cells overexpressing CFIm25 and control were treated with PMA for 0, 6 and 24 hours and live cells visualized and counted by the Trypan blue exclusion assay with respect to control. The graph presents the percentage of cells that are suspended or attached at each time point. The viability of cells for OE-CFIm25 with respect to OE-control after differentiation (*right*) indicates the amount of resazurin converted to resorufin, which is proportional to the number of viable cells in the sample, compared to those in the absence of PMA. The figure represents mean ± SE from three independent experiments. **(C & D)** For cell cycle analysis, flow cytometry data was obtained for HL-60 **(C)** and THP-1 **(D)** cells overexpressing CFIm25 and treated with PMA for indicated hours with respect to control, where the Y axis represents cell numbers, and the X axis represents incorporation of propidium iodide (PI). Data is representative of at least three biological replicates. **(E and F)** CD38 levels in genetically manipulated HL-60 **(E)** and THP-1 **(F)** cells by flow cytometry. Flow cytometry staining of the surface marker CD38 at different time points during differentiation of control cells and cells overexpressing CFIm25, where the percentage on the Y axis represents cells positive for CD38, with FSC on the X axis. Data is representative of at least three biological replicates.

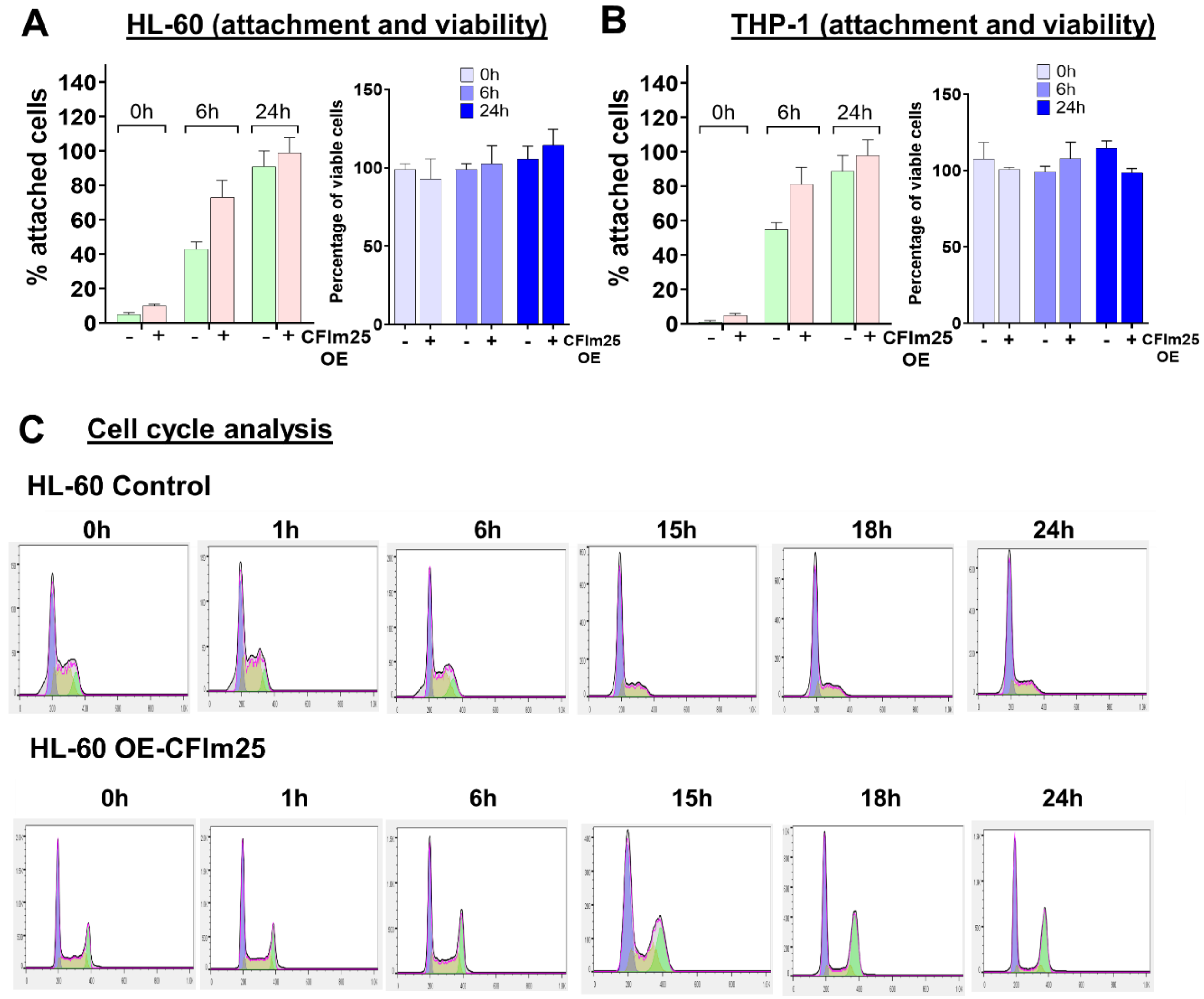

Supplementary Fig 2D-F

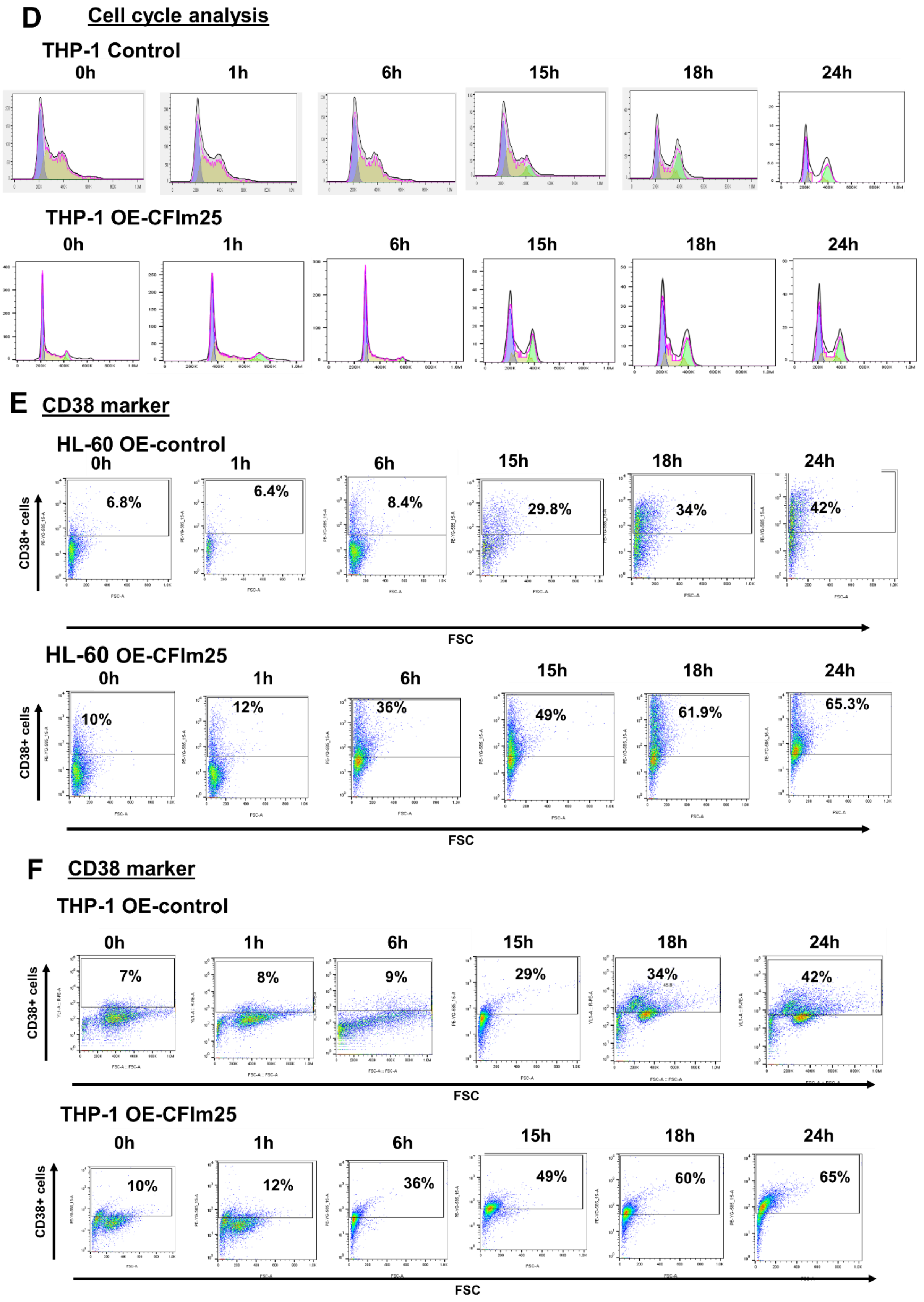

Supplementary Fig 3. Effect of CFIm25 knockdown. (A and B) Attachment and viability assays for (A) HL-60 and (B) THP-1 control cells or cells knocked down with siRNA against CFIm25. For the attachment assay (*left*), cells treated with siRNA against CFIm25 or control were treated with PMA for 24 hours and live cells visualized and counted by the Trypan blue exclusion assay. The graph presents the percentage of cells that are suspended or attached. Viability was determined using the resazurin as described in Sup. Fig. 2. The figure represents mean ± SE from three independent experiments. **(C and D) Raw data for cell cycle analysis.** Flow cytometry data of HL-60 **(A)** and THP-1 **(B)** cells knocked down for CFIm25 and treated with PMA for indicated hours with respect to control, where the Y axis represents cell numbers, and the X axis represents incorporation of propidium iodide (PI). Data is representative of at least three biological replicates. **(E and F) CD38 levels in genetically manipulated HL-60 (E) and THP-1 (F) cells by flow cytometry.** Flow cytometry staining of surface marker CD38 at different time points during differentiation of control cells and cells knocked down with siRNA against CFIm25, where the percentage on the Y axis represents cells positive for CD38, with FSC on the x-axis. Data are representative of at least three biological replicates.

Supplementary Fig 3A-3D

A **HL-60 (attachment and viability)**

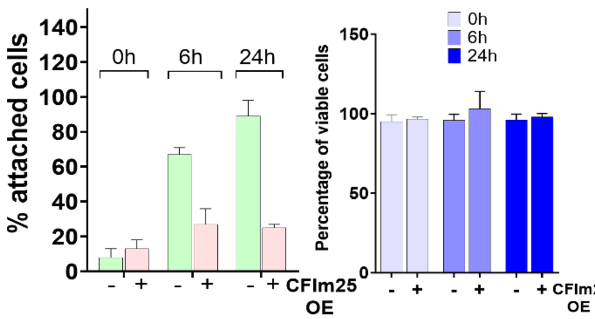

B **THP-1 (attachment and viability)**

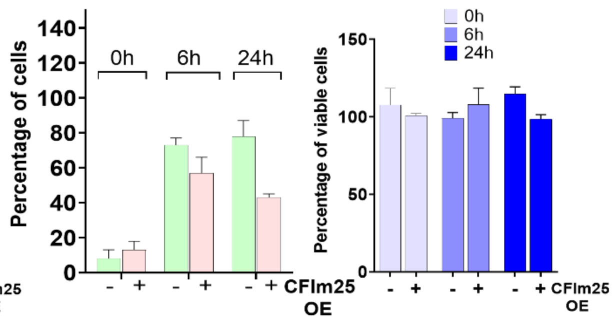

C **Cell cycle analysis**

HL-60 si-control

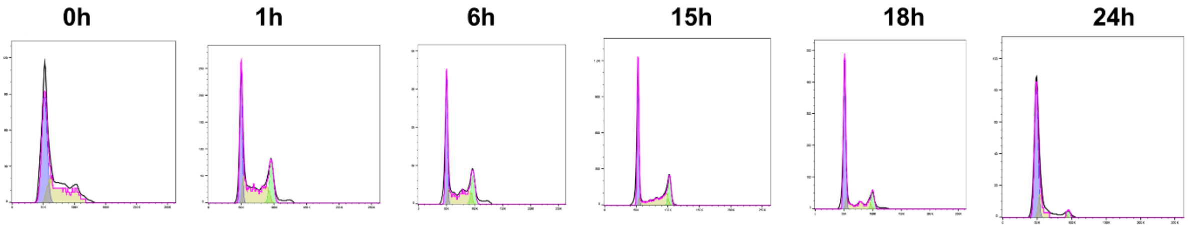

HL-60 si-CFIm25

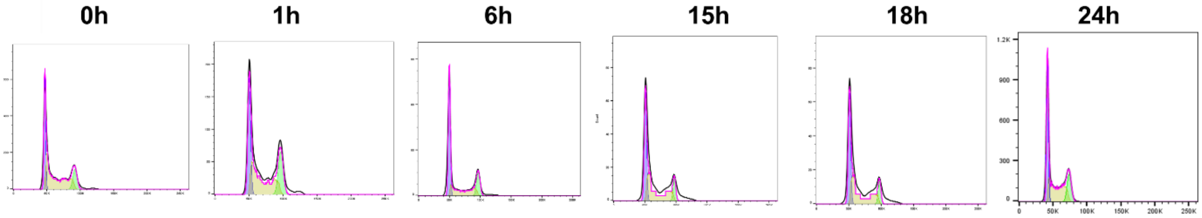

D **Cell cycle analysis**

THP-1 si-control

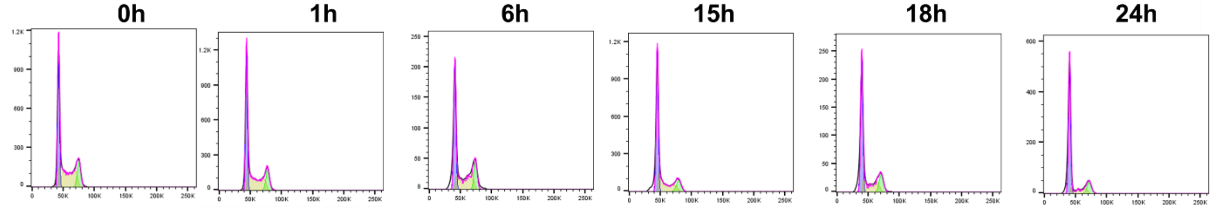

THP-1 si-CFIm25

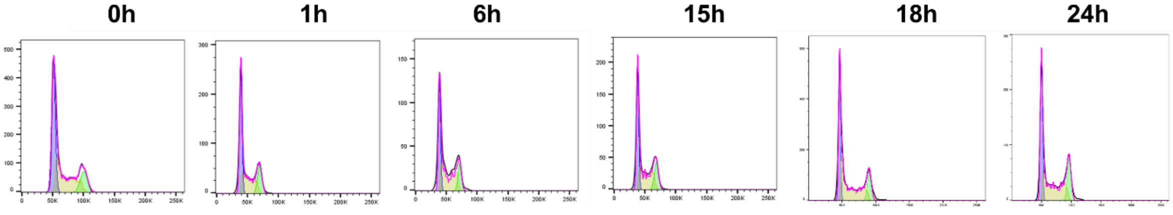

Supplementary Fig 3E-3F

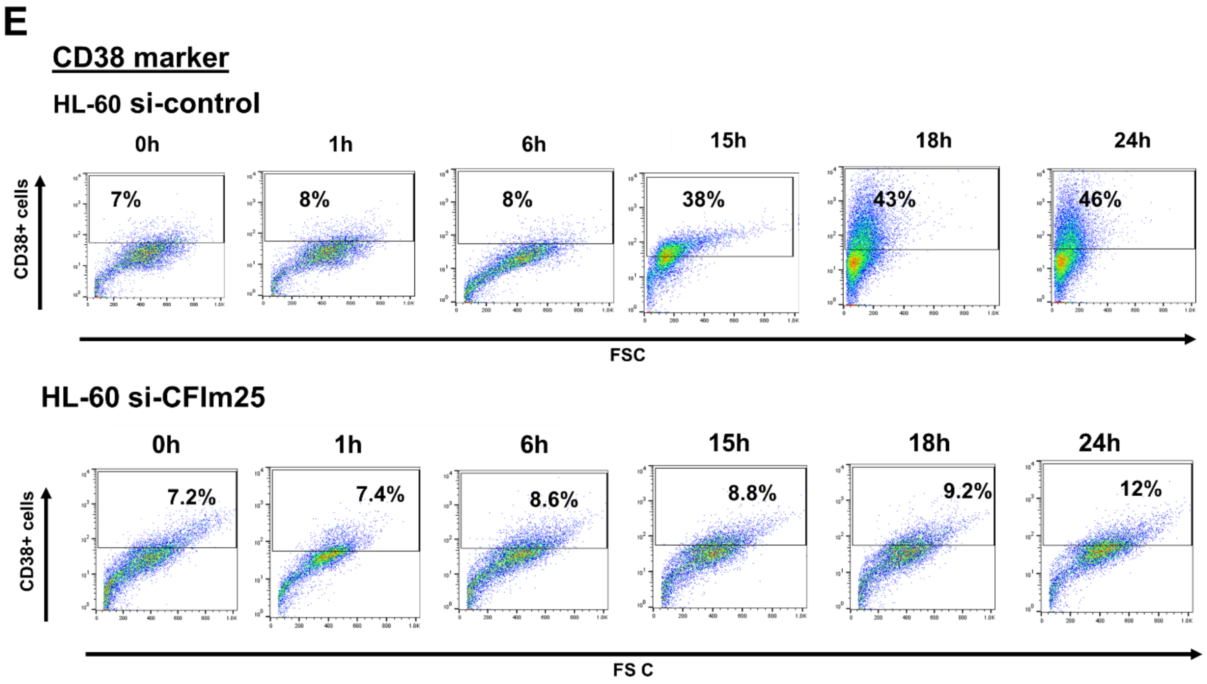

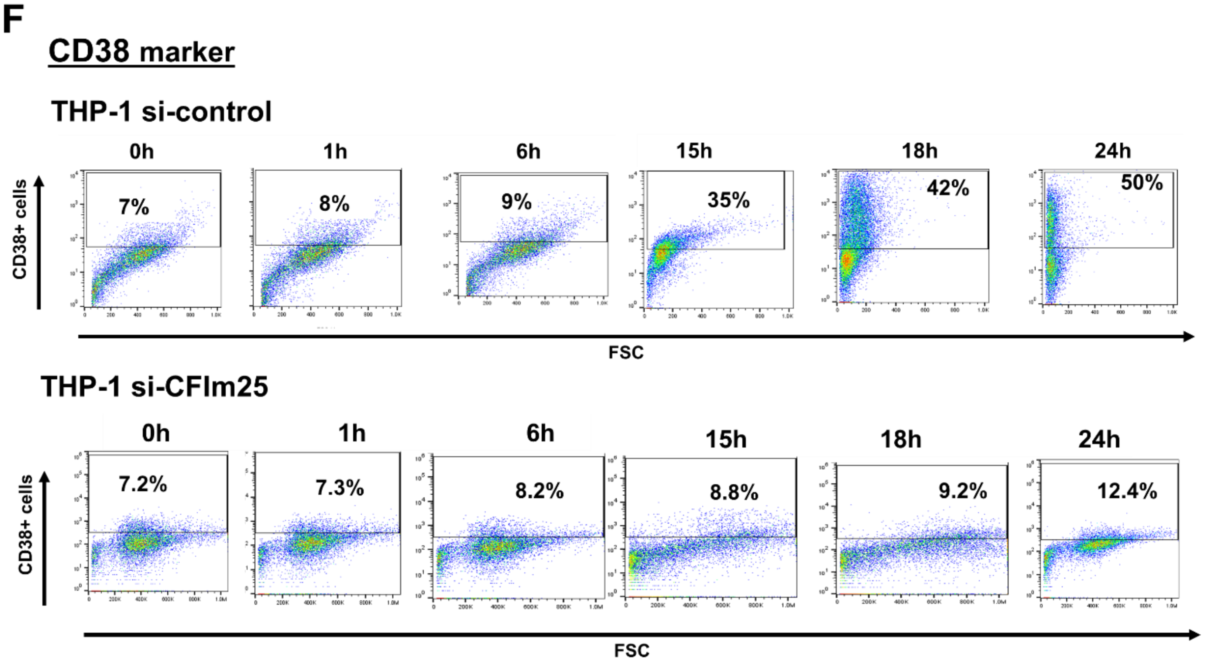

Supplementary Figure 4. Expression of CCND1 long mRNA isoforms after CFIm25 manipulation. RT-qPCR–based analysis of the levels of the long isoforms of mRNAs after overexpression or knockdown of CFIm25 and treatment with PMA for 0-24h in HL-60 **(A)** and THP-1 **(B)** cells. The analysis was performed as described in Fig. 4 using a primer upstream of the distal PAS. P value <0.05 was considered significant, where * = $P \leq 0.05$; **= $P \leq 0.01$.

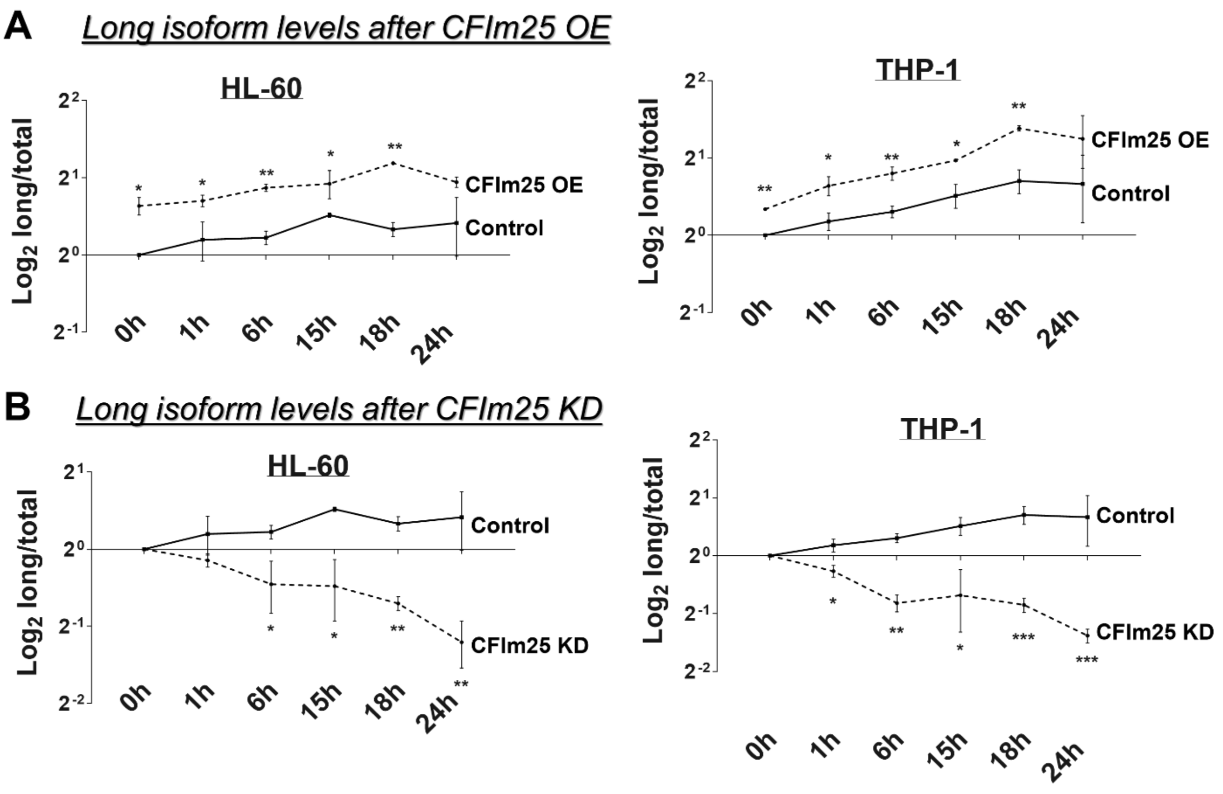

Supplementary Figure 5. Changes in poly(A) site usage after 6 hours of PMA treatment in control HL-60 cells compared to those overexpressing CFIm25. (A and B) UCSC genome browser plots of merged RNA sequencing tracks highlighting the 3′-UTR profile differences for lengthened genes **(A)** or shortened genes **(B)** after 6 hours of PMA treatment of HL-60 cells overexpressing CFIm25 with respect to control for three biological replicates. The colors of the tracks represent OE-control (red) and OE-CFIm25 (blue). Proximal (P) and distal (D) PASs are indicated with arrows and chromosome co-ordinates and distance between the differentially used PASs are indicated at the top of each plot. The track labeled Annotated Poly(A) Sites gives the positions of known PASs from the polyA_DB database (Version V4.1s) identified in a variety of human cell types. For each of the browser plots, the Y axis value is reads per million (RPM) and the right side displays quantitative bar graphs reflecting the change in proximal PAS usage with respect to the total read counts. An unpaired t-test was performed to determine the significance between the treatment groups, and P value <0.05 was considered significant.

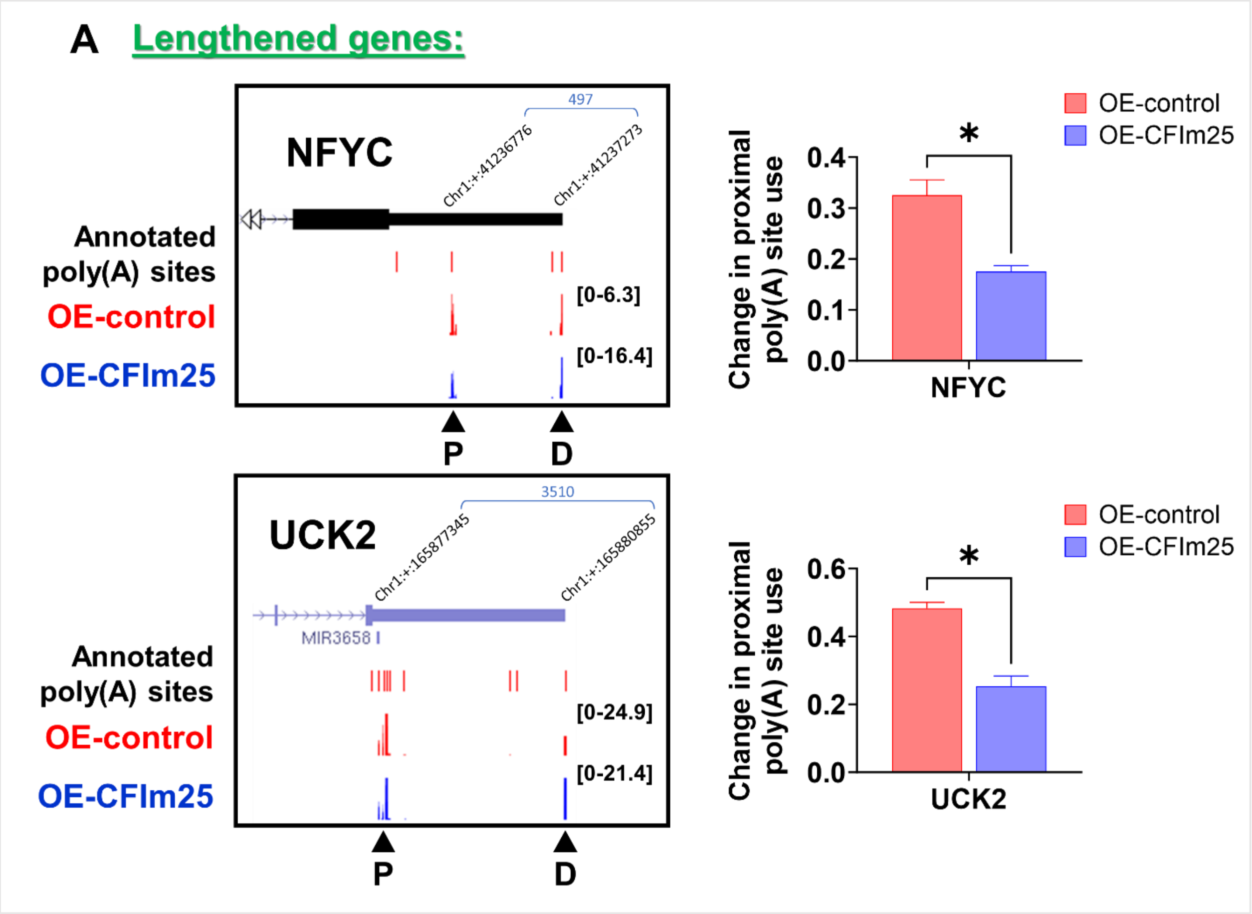

Supplementary Figure 5

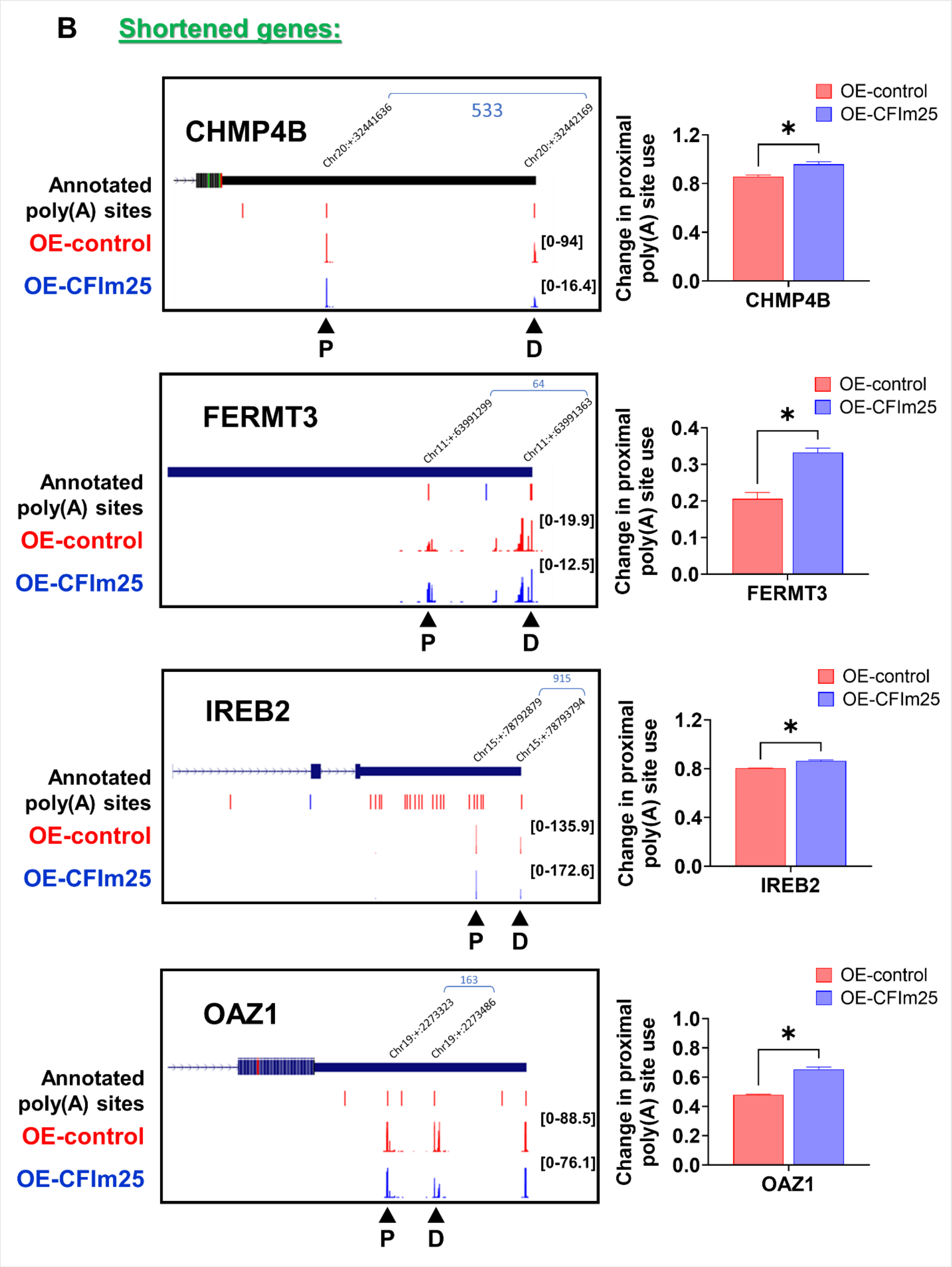

Supplementary Figure 5

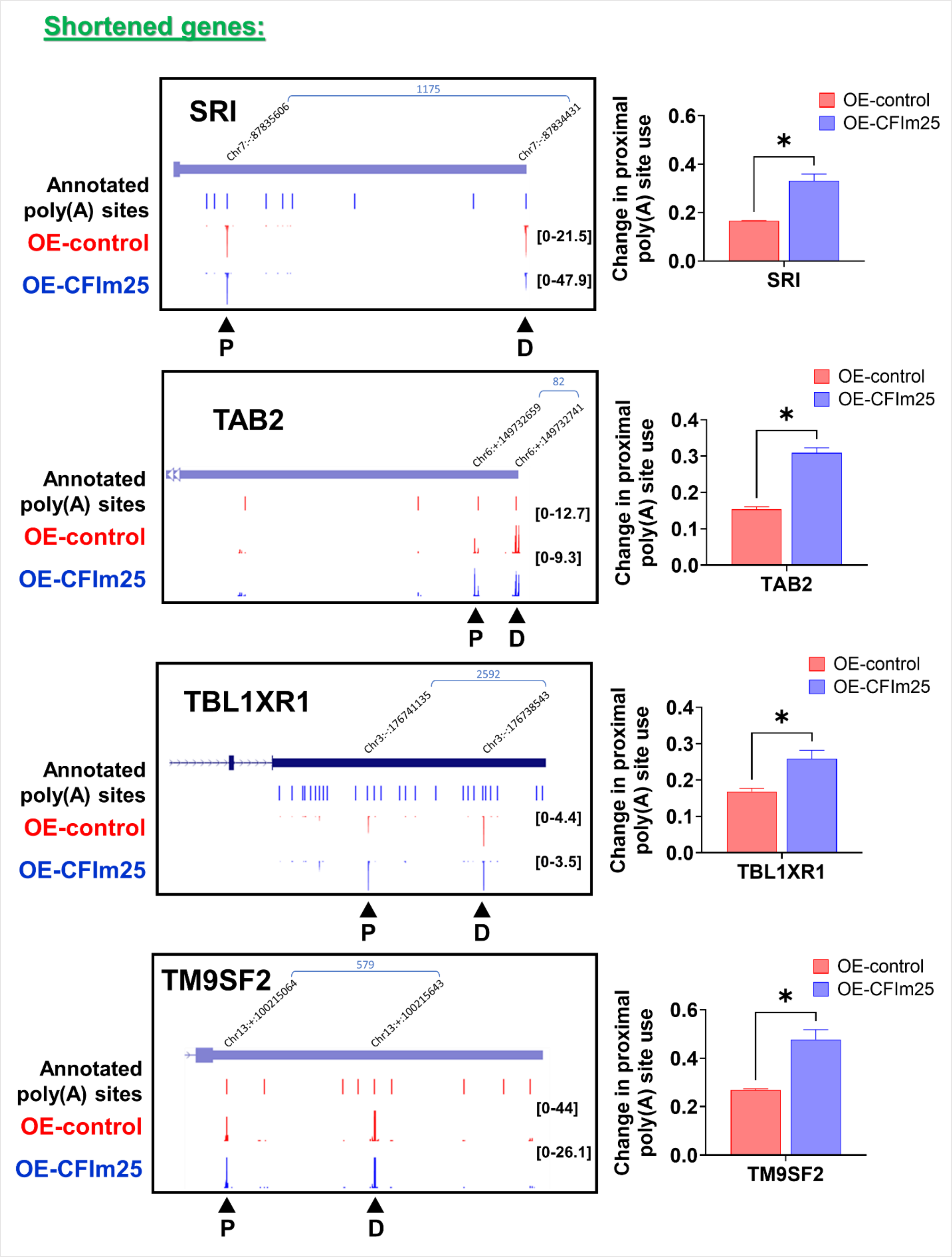

Supplementary Figure 6. CFIm25 overexpression results in APA changes of specific genes and altered protein expression in PMA-treated THP-1 cells. (A) APA and expression analysis of targets. RT-qPCR–based analysis of the expression (upper panel) and distal PAS usage (lower panel) of different genes in THP-1 cells revealed by 3'Quant-seq as lengthened genes (NFYC, UCK2) or shortened genes (CHMP4B, FERMT3, IREB2, OAZ1, SRI, TAB2, TBL1XR1 and TM9SF2). The analysis was performed as described in Fig. 4. P value <0.05 was considered significant, where * = $P \leq 0.05$; **= $P \leq 0.01$; *** = $P \leq 0.001$. **(B) Protein levels of genes with APA changes.** Western blot analysis of proteins encoded by the shortened and lengthened genes as uncovered by 3'Quant-seq in THP-1 cells overexpressing CFIm25 with respect to control. GAPDH serves as the loading control.

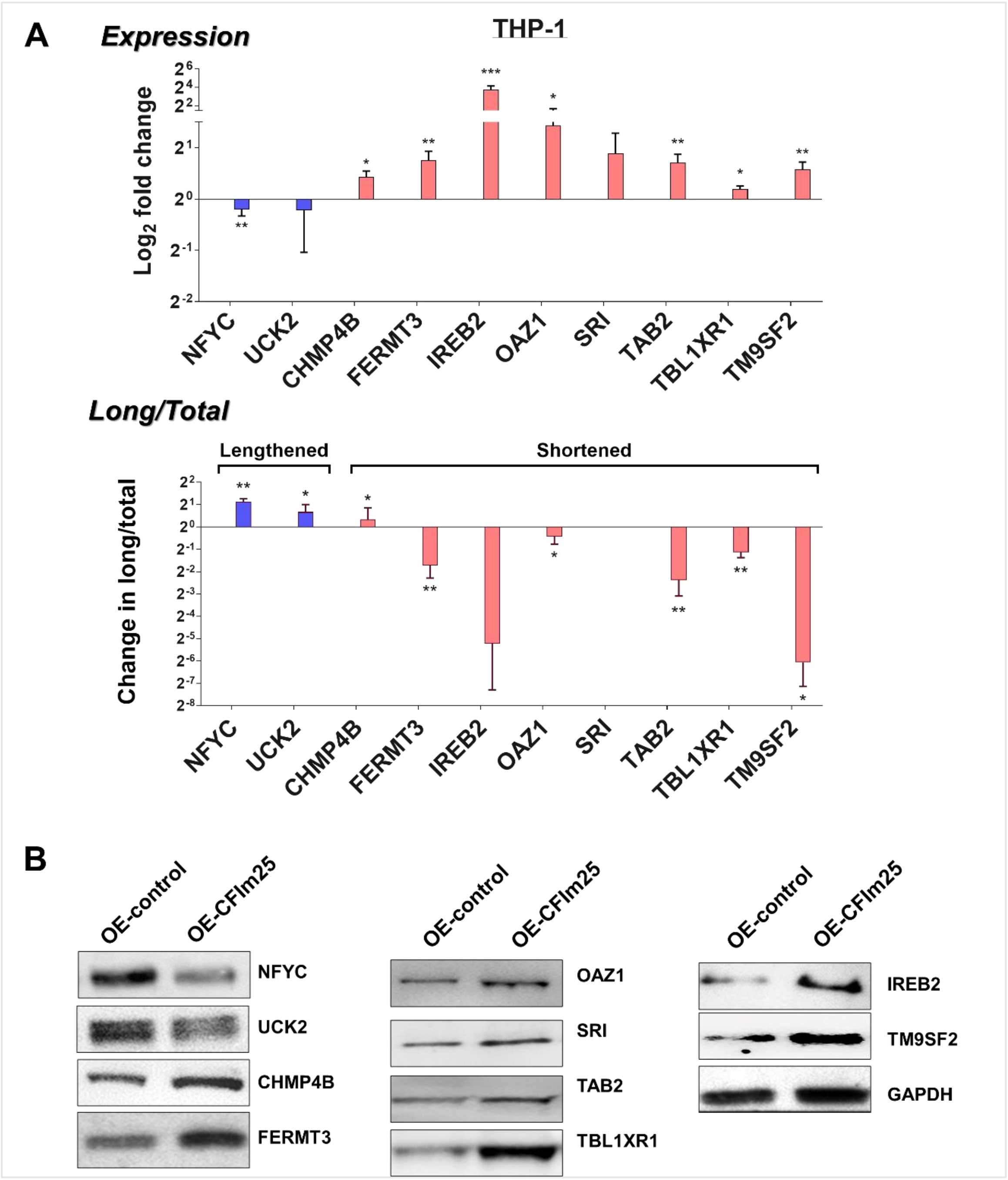

Supplementary Figure 7. Changes in long isoform levels of the NF-κB pathway components after CFIm25 overexpression and APA changes of these mRNAs during differentiation of primary monocytes. RT-qPCR–based analysis of the levels of the long isoforms of NFKB1, TAB2 and TBL1XR1 mRNAs after overexpression of CFIm25 and treatment with PMA for 0-24h in HL-60 **(A)** and THP-1 **(B)** cells. The analysis was performed as described in Fig. 4 using a primer upstream of the distal PAS. P value <0.05 was considered significant, where * = $P \leq 0.05$; **= $P \leq 0.01$. **(C)** Change in the long/total mRNA ratio for TAB2, TBL1XR1 and NFKB1 mRNAs in primary monocytes after 7 days of exposure to M-CSF.

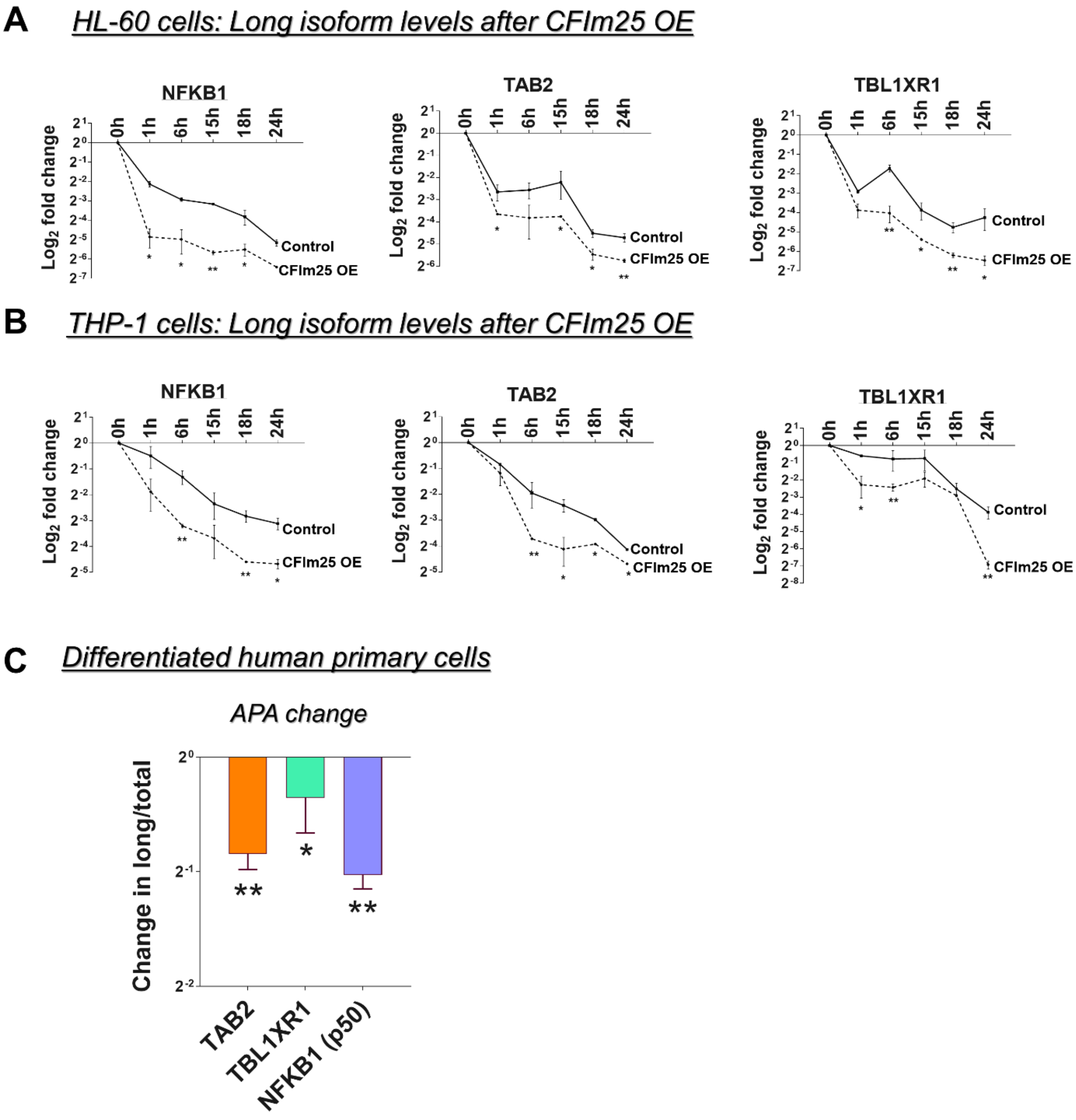

Supplementary Figure 8. CFIm25 depletion favors expression of the long isoforms of effectors of the NF-κB pathway. RT-qPCR–based analysis of the change in distal PAS usage indicated as the long/total ratio **(A and D)** and the expression of total **(B and E)** and long isoforms **(C and F)** of NFKB1, TAB2 and TBL1XR1 mRNAs after overexpression of CFIm25 and treatment with PMA for 0-24h in HL-60 **(A, B, C)** and THP-1 **(D, E, F)** cells. The analysis was performed as described in Fig. 4.

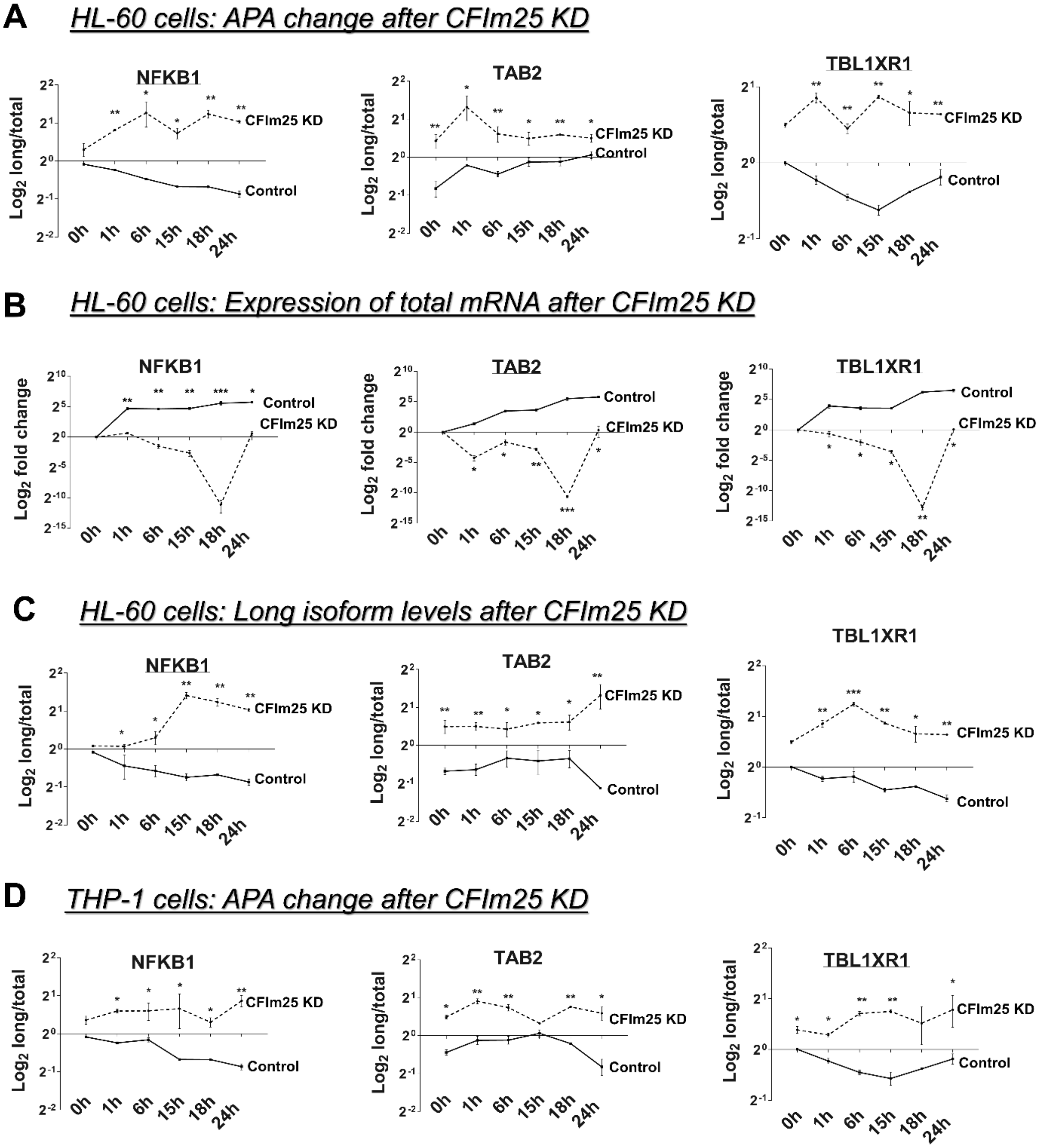

Supplementary Figure 8 (E-F)

E *THP-1 cells: Expression of total mRNA after CFIm25 KD*

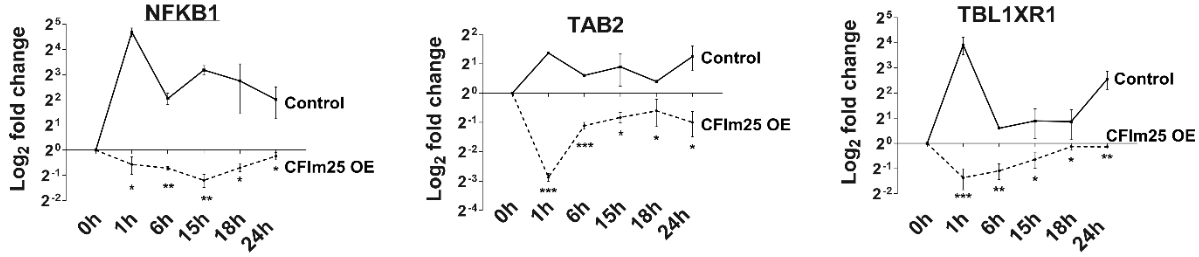

F *THP-1 cells: Long isoform levels after CFIm25 KD*

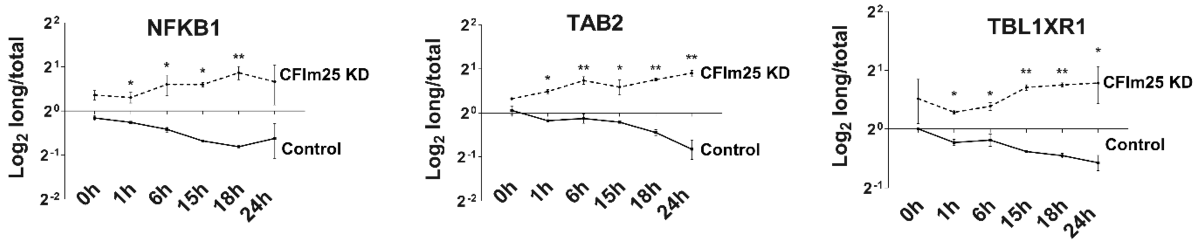

Supplementary Figure 9. TAB2 and TBL1XR1 mediate CFIm25-induced NF-κB activation. Representative raw flow cytometry data in HL-60 (A) and THP-1 (B) cells overexpressing CFIm25, followed by treatment with control siRNAs or siRNA against TAB2 or TBL1XR1 and induction of differentiation with PMA for 24 hours.

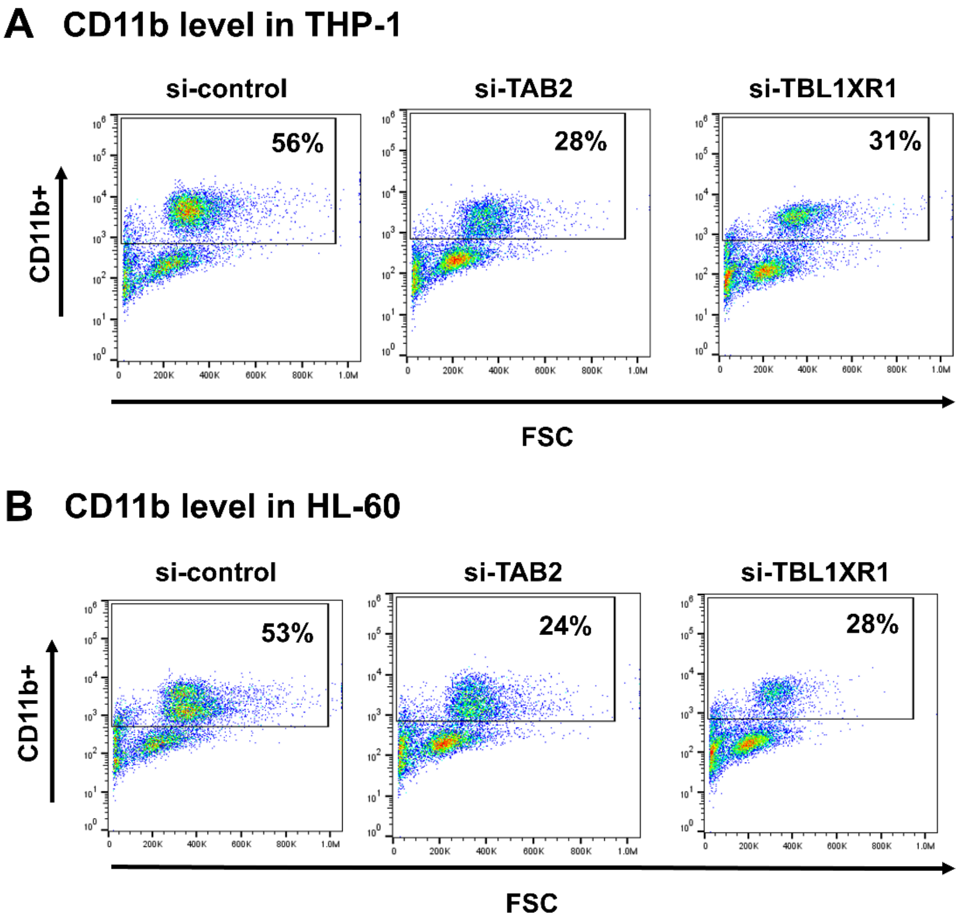

Supplementary Figure 10. Representative raw flow cytometry data showing that NF-κB pathway inhibitor BI605906 affects CD38 (A) and CD11b (B) levels to a lesser extent in HL-60 or THP-1 cells overexpressing CFIm25 during differentiation. The percentage represents cells positive for CD38 or CD11b. Gating for both is performed according to Supplementary Fig. 1C and D.

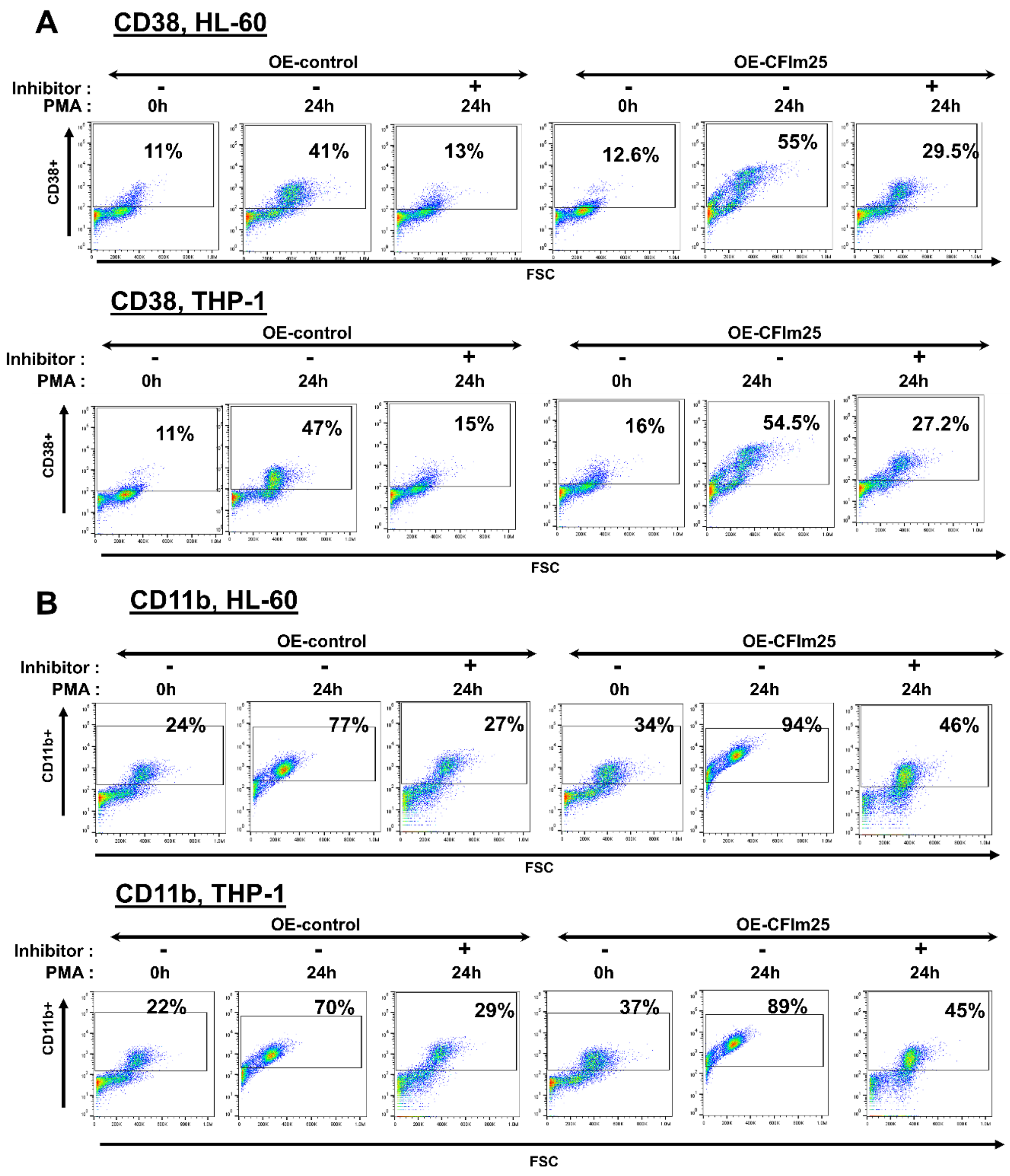

Supplementary Figure 11. CFIm25 overexpression provides resistance to BAY 11-7082. **(A)** Attachment assay. HL-60 and THP-1 control cells or cells overexpressing CFIm25 were incubated with or without BAY 11-7082, and then treated with PMA for 0 and 24 hours, and the percentage of cells attached at each time point determined. **(B)** Viability assay. The viability of cells was determined by the resazurin conversion assay. **(C and D)** Effect on macrophage markers CD38 and CD11b by flow cytometry of HL-60 and THP-1 control cells or those overexpressing CFIm25 with or without treatment with NF-κB inhibitor. The expression of CD38 and CD11b is shown as a percentage of CD38+ or CD11b+ cells. **(E and F)** Representative raw flow cytometry data showing that BAY 11-7082 affects CD38 **(E)** and CD11b **(F)** levels to a lesser extent in HL-60 or THP-1 cells overexpressing CFIm25 during differentiation. The percentage represents cells positive for CD38 or CD11b. Gating for both is performed according to Supplementary Fig. 1C and D. Data is representative of at least three biological replicates and is plotted as mean ± SE. P value <0.05 was considered significant, where * = P ≤ 0.05; *** = P ≤ 0.001.

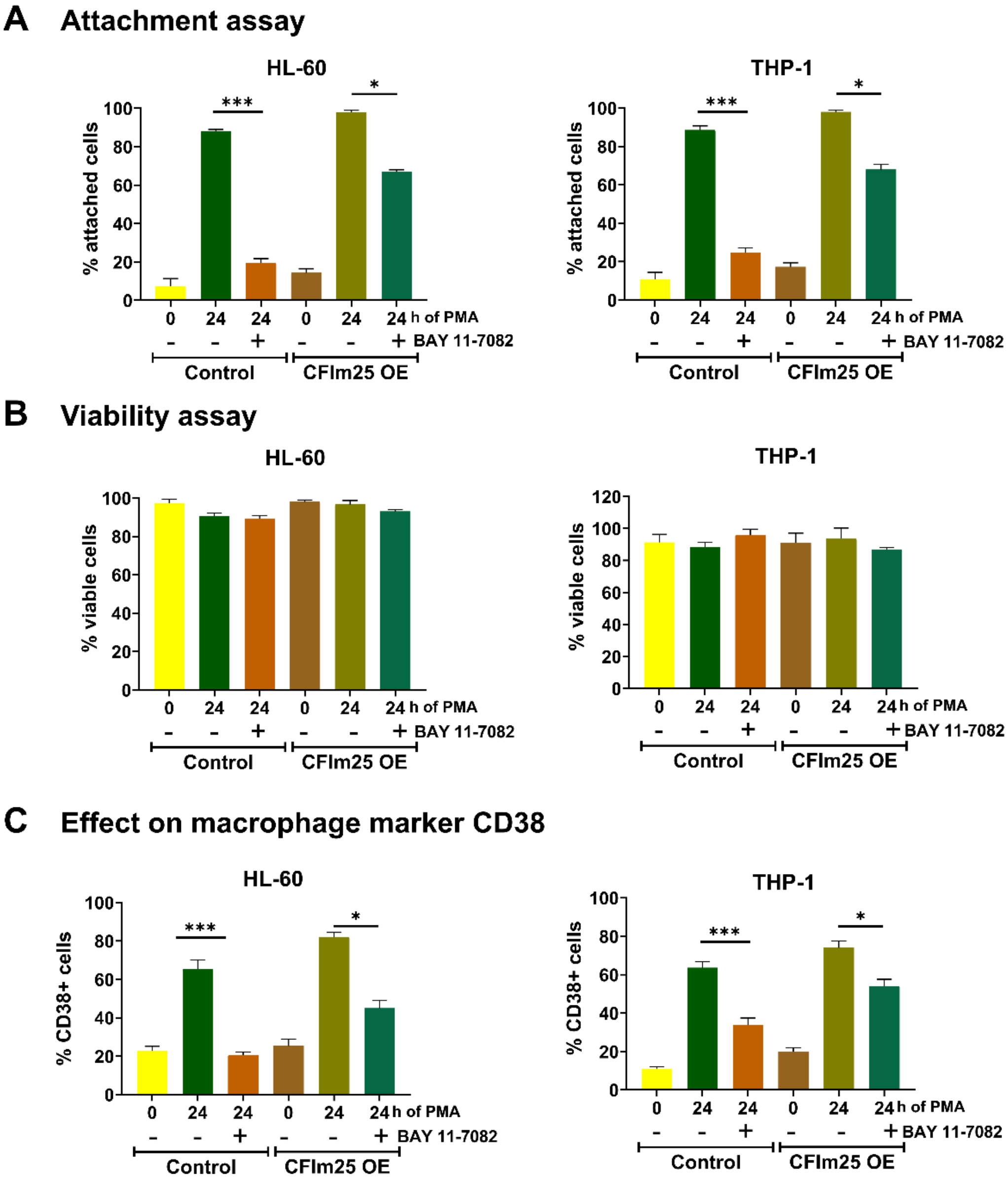

Supplementary Figure 11 (D-F)

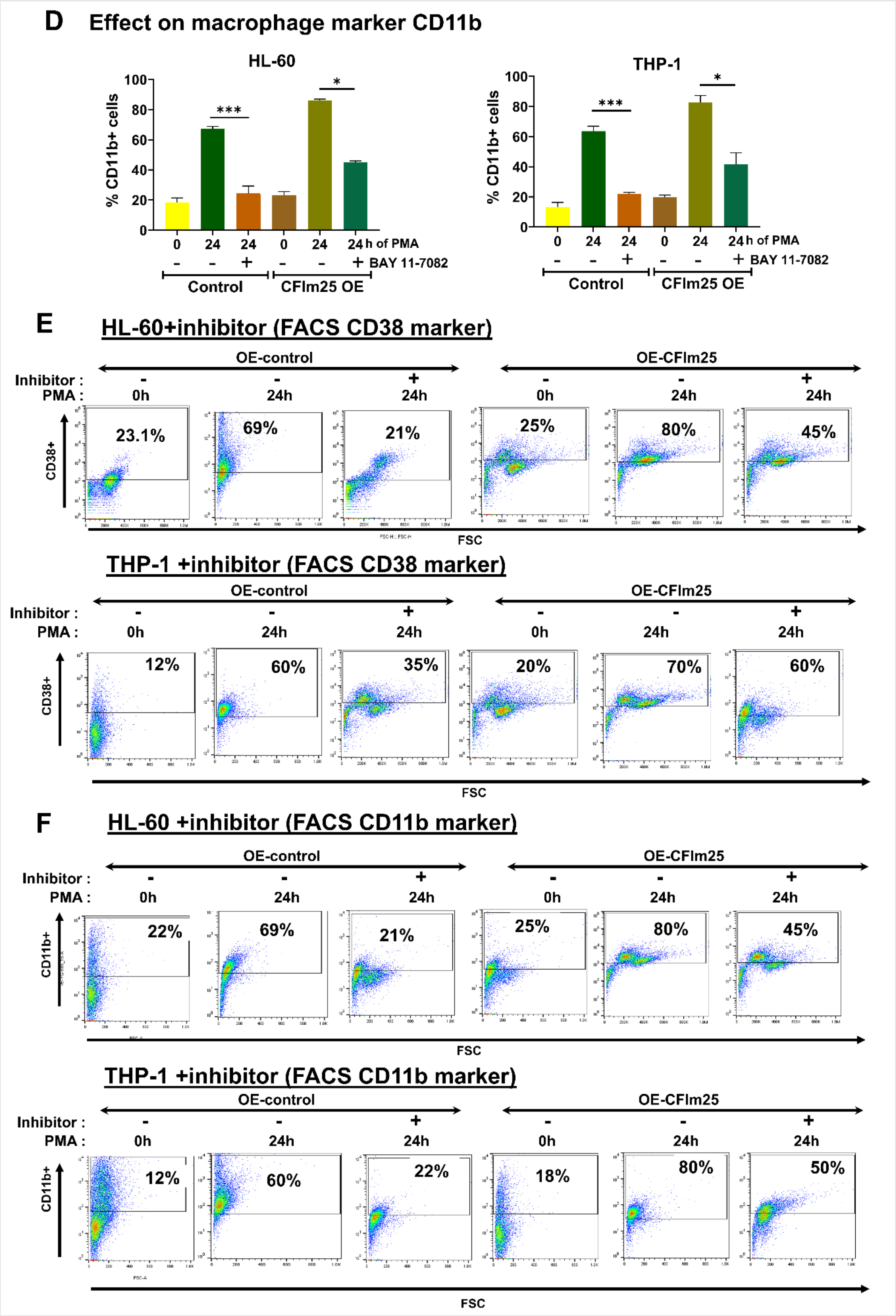

Supplement: Supplementary file 1 — Supplementary Material 1 [file 12964_2025_2114_MOESM1_ESM.pdf]
